# Metabolic profiling of *Lantana camara* L. using UPLC-MS/MS and revealing its inflammation-related targets using network pharmacology-based and molecular docking analyses

**DOI:** 10.1038/s41598-022-19137-0

**Published:** 2022-09-01

**Authors:** Alaa A. El-Banna, Reham S. Darwish, Doaa A. Ghareeb, Abdelrahman M. Yassin, Shaymaa A. Abdulmalek, Hend M. Dawood

**Affiliations:** 1 Department of Pharmacognosy, Faculty of Pharmacy, Alexandria University, Alexandria, 21521 Egypt; 2 Center of Excellence for Drug Preclinical Studies (CE-DPS), Pharmaceutical and Fermentation Industry Development Center, City of Scientific Research & Technological Applications, New Borg El Arab, Alexandria Egypt; 3 Bio-Screening and Preclinical Trial Lab, Biochemistry Department, Faculty of Science, Alexandria University, Alexandria, Egypt; 4 Biochemistry Department, Faculty of Science, Alexandria University, Alexandria, Egypt

**Keywords:** Computational biology and bioinformatics, Plant sciences, Diseases

## Abstract

*Lantana camara* L. is widely used in folk medicine for alleviation of inflammatory disorders, but studies that proved this folk use and that revealed the molecular mechanism of action in inflammation mitigation are not enough. Therefore, this study aimed to identify *L. camara* phytoconstituents using UPLC-MS/MS and explain their multi-level mechanism of action in inflammation alleviation using network pharmacology analysis together with molecular docking and in vitro testing. Fifty-seven phytoconstituents were identified in *L. camara* extract, from which the top hit compounds related to inflammation were ferulic acid, catechin gallate, myricetin and iso-ferulic acid. Whereas the most enriched inflammation related genes were PRKCA, RELA, IL2, MAPK 14 and FOS. Furthermore, the most enriched inflammation-related pathways were PI3K-Akt and MAPK signaling pathways. Molecular docking revealed that catechin gallate possessed the lowest binding energy against PRKCA, RELA and IL2, while myricetin had the most stabilized interaction against MAPK14 and FOS. In vitro cytotoxicity and anti-inflammatory testing indicated that *L. camara* extract is safer than piroxicam and has a strong anti-inflammatory activity comparable to it. This study is a first step in proving the folk uses of *L. camara* in palliating inflammatory ailments and institutes the groundwork for future clinical studies.

## Introduction

Inflammation is a complex process, triggered by injury, infection, or genetic alterations, leading to signaling cascades stimulation, transcription factors activation, gene expression, elevated levels of inflammatory enzymes, and liberation of diverse oxidants and pro-inflammatory mediators in inflammatory cells. Immoderate levels of oxidants and inflammatory molecules are harmful to normal tissue. They can cause toxicity, lack of barrier function, anomalous cell proliferation, impeding normal function of tissues and organs and eventually resulting in systemic disorders^1^. The ordinary inflammation treatment mostly includes steroidal and non-steroidal anti-inflammatory drugs and opiates. These agents have many adverse effects such as gastric ulcers, tolerance, and dependence^2^. Therefore, the attention is now paid to natural products with the aim to gain more effective anti-inflammatory agents with less or no side effects. Recently, many medicinal plants have been successfully used for alleviation of inflammation as they exhibited significant anti-inflammatory activities, such as macrophage differentiation, lymphocyte activation, and propagation of apoptosis^3^. A remarkable example of these medicinal plants is *Lantana camara* L. (Verbanaceae)^2,4^.

*Lantana camara* L. is a flowering plant belongs to family Verbanaceae, also called wild or red sage. It is indigenous to Africa and tropical America, and it is the most prevalent species of genus *Lantana*. It is considered as a medicinal aromatic plant, a notorious weed and as a common ornamental garden plant^4^. It is used in folk medicine in alleviation of several inflammatory ailments, such as rheumatism, swellings, bronchitis, and asthma^5^. In addition, it is used as an adjuvant therapy in mitigation of cancers, chicken pox, measles, eczema, tumors, high blood pressure, bilious fevers, and catarrhal infections^6^. Furthermore, it is utilized to palliate malaria, tuberculosis, lymphadenitis, mumps, stomachache, and bone pain^7^.

Former in vivo and in vitro studies of *L. camara* showed that it exerts its anti-inflammatory activity through suppressing several inflammatory mediators such as COX-2, LOX, NO, ROS, NF-kB, or inhibition of inflammatory signals transmission^4^. Moreover, *L. camara* extract showed an inhibition to edema induced by carrageenan^2,8^, and it was found to suppress iNOS which has an important role in inflammation^9^.

The plant extracts have a complicated nature which makes it difficult to explain their molecular mechanisms of action. This may be because the plant extracts can act on several targets at the same time, or due to the synergism between their chemical constituents^10^. Recently, the molecular targets and the related disease pathways of plant constituents have been successfully predicted via the application of network pharmacology-based analysis. This technique allows to envisage compound-target-gene-disease network hence, facilitating the projection of the multi-target mechanism of plant extracts^11,12^. Network pharmacological analysis has been successfully used to explain the mechanism of action of many medicinal plants in alleviating different diseases^13–17^.

**Figure 1:**
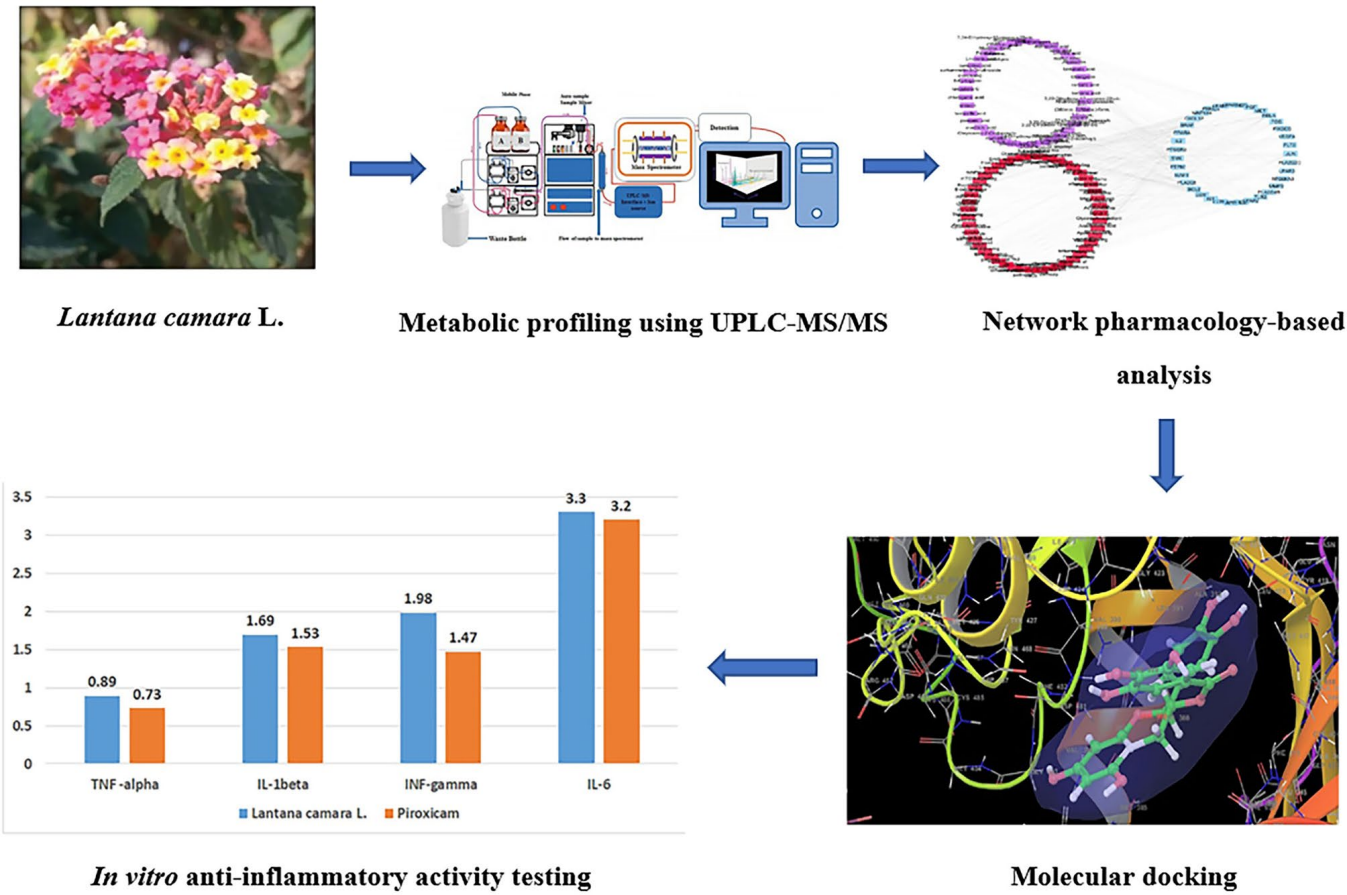
Study workflow diagram.

Although there are some studies in literature that explain the mechanisms of action of *L. camara* in mitigating inflammatory disorders^2,4,8,9,18–20^, this work was carried out to allow for more in-depth understanding of these molecular mechanisms of action using network pharmacology-based and molecular docking analyses, in addition to in vitro studies, thus proving the folk uses of *L. camara* as anti-inflammatory natural product. The workflow of this study is illustrated in Fig. 1.

## Results

### UPLC-MS/MS analysis of *L. camara* extract

The base peak chromatogram of *L. camara* extract showed the presence of 57 metabolites (Supplementary Fig. S1) which were recognized through comparing their retention times to external standards, CRC, Wiley, reference literature in addition to our in-house database. The UPLC-MS/MS metabolic profile was presented in Table 1.

**Table 1:** Metabolites identified in the extract of *L. camara* using UPLC-MS/MS in negative ionization mode.

Peak number	Retention time (min)	Identified compounds	M−H^a^	Molecular weight	Chemical class	Element composition	MS^n^ fragments^b^
1	1.14	1-cinnamoyl rhamnoside	293	294	Aromatic acid glycoside	C_15_H_18_O_6_	148
2	1.26	Gallic acid	169	170	Phenolic acid	C_7_H_6_O_5_	125, 107
3	1.46	Chlorogenic acid	353	354	Phenolic acid	C_16_H_18_O_9_	191, 179
4	1.67	Ferulic acid	193	194	Phenolic acid	C_10_H_10_O_4_	149, 175
5	2.16	Isoferulic acid	193	194	Phenolic acid	C_10_H_10_O_4_	149, 175
6	4.72	Cinnamic acid	147	148	Aromatic monocarboxylic acid	C_9_H_8_O_2_	103, 129
7	5.55	2-Oxoisocaproate	129	130	Short-chain keto acids	C_6_H_10_O_3_	115, 100, 70
8	7.26	Catechin gallate	441	442	Flavonoid gallic acid ester	C_22_H_18_O_10_	109, 121, 271, 289
9	8.35	Isorhamnetin-3-O-rutinoside	623	624	Flavonoid	C_28_H_32_O_16_	315, 300, 271, 255
10	8.85	Pectolinarin	621	622	Flavonoid	C_29_H_34_O_15_	314, 299, 284, 234
11	9.70	verbascoside	623	624	Phenylethanoid glycoside	C_29_H_36_O_15_	179, 161, 461, 315, 135
12	10.95	isoverbascoside	623	624	Phenylethanoid glycoside	C_29_H_36_O_15_	179, 161, 461, 315, 135
13	11.34	Theveside	389	390	Iridoid glycosides	C_16_H_22_O_11_	227, 345, 371
14	12.07	Geniposide	387	388	Iridoid glycosides	C_17_H_24_O_10_	225, 207, 123, 101
15	12.44	8-epiloganin	389	390	Iridoid glycosides	C_17_H_26_O_10_	359, 227, 329, 311
16	12.72	Chrysoeriol-O-hexoside	461	462	Flavonoid	C_22_H_22_O_11_	299, 284
17	13.58	Rhamnocitrin-O-glucoside	461	462	Flavonoid	C_22_H_22_O_11_	299, 446
18	13.84	Linaroside	475	476	Flavonoid	C_23_H_24_O_11_	313, 460, 445
19	14.23	Durantoside I	551	552	Iridoid glycosides	C_26_H_32_O_13_	389, 521, 491
20	15.88	Scoparin(Chrysoeriol 8-C-glucoside)	461	462	Flavonoid	C_22_H_22_O_11_	371, 341, 298
21	16.05	6- Methoxy-5-hydroxynaphtho[2,3-b]furan-4,9-dione	243	244	Furanonaphthoquinone	C_13_H_8_O_5_	228, 215, 187
22	16.13	Afzelechin	273	274	Flavonoid	C_15_H_14_O_5_	257, 137
23	20.90	Myricetin	317	318	Flavonoid	C_15_H_10_O_8_	151,179
24	23.06	Kaempferol	285	286	Flavonoid	C_15_H_10_O_6_	239, 187,143
25	24.21	Chrysoeriol	299	300	Flavonoid	C_16_H_12_O_6_	284, 255
26	24.48	Cirsiliol	329	330	Flavonoid	C_17_H_14_O_7_	314, 299, 285, 271
27	25.15	pectolinarigenin	313	314	Flavonoid	C_17_H_14_O_6_	299, 284, 234
28	26.32	Penduletin	343	344	Flavonoid	C_18_H_16_O_7_	328, 313, 298
29	26.89	3',4'-Dimethoxy-7-hydroxyflavanone	297	298	Flavonoid	C_17_H_14_O_5_	284, 254, 135
30	26.98	Lamiide	421	422	Terpene glycoside	C_17_H_26_O_12_	391, 259, 361, 343
31	27.97	3,22,24-Trihydroxy-12-oleanen-28-oic acid; (3β,22β)-form, 3-Ketone	485	486	Oleane-type triterpene	C_30_H_46_O_5_	467, 441
32	28.35	3,12,13-Trihydroxy-28-oleananoic acid; (3β,12β,13β)-form, 3-ketone	487	488	Oleane-type triterpene	C_30_H_48_O_5_	443, 469
33	28.68	3,24-Dioxo-12-oleanen-28-oic acid	467	468	Oleane-type triterpene	C_30_H_44_O_4_	423
34	28.72	24-Hydroxy-3-oxo-12-oleanen-28-oic acid	469	470	Oleane-type triterpene	C_30_H_46_O_4_	451, 425
35	28.80	Lantanolic acid	469	470	Oleane-type triterpene	C_30_H_46_O_4_	421, 391, 420, 377
36	28.83	Icterogenin	567	568	Oleane-type triterpene	C_35_H_52_O_6_	451, 407, 98
37	28.87	Lantanilic acid	567	568	Oleane-type triterpene	C_35_H_52_O_6_	451, 407, 98
38	28.94	Camaric acid	567	568	Oleane-type triterpene	C_35_H_52_O_6_	451, 407, 98
39	28.97	22-Tigloyloxylantanolic acid	567	568	Oleane-type triterpene	C_35_H_52_O_6_	549, 523
40	29.00	Lantadene A	551	552	Oleane-type triterpene	C_35_H_52_O_5_	98, 507
41	29.19	Lantadene B	551	552	Oleane-type triterpene	C_35_H_52_O_5_	98, 435, 391
42	29.32	Dihydrorehmannic acid	553	554	Oleane-type triterpene	C_35_H_54_O_5_	535, 509
43	30.28	Lantoic acid	485	486	Ursane-type triterpene	C_30_H_46_O_5_	437, 421, 407
44	30.78	3,24-Dioxo-12-ursen-28-oic acid	467	468	Ursane-type triterpene	C_30_H_44_O_4_	423
45	31.20	24-Hydroxy-3-oxo-12-ursen-28-oic acid	469	470	Ursane-type triterpene	C_30_H_46_O_4_	451, 425
46	31.37	3,25-Epoxy-3-hydroxy-12-ursen-28-oic acid	469	470	Ursane-type triterpene	C_30_H_46_O_4_	451, 425
47	31.43	Pomonic acid	469	470	Ursane-type triterpene	C_30_H_46_O_4_	451, 407
48	31.55	Lantic acid	469	470	Ursane-type triterpene	C_30_H_46_O_4_	421, 391, 420, 377
49	31.64	Pomolic acid	471	472	Ursane-type triterpene	C_30_H_48_O_4_	453, 411
50	31.73	Camarinic acid	527	528	Ursane-type triterpene	C_32_H_48_O_6_	58, 451, 407
51	31.82	Ursoxy acid	483	484	Ursane-type triterpene	C_31_H_48_O_4_	453, 439
52	31.90	Lantacin	569	570	Ursane-type triterpene	C_35_H_54_O_6_	98, 453, 409
53	34.54	Myristoleic acid	225	226	Unsaturated fatty acid	C_14_H_26_O_2_	54, 181, 207
54	36.57	Linolenic acid	277	278	Unsaturated fatty acid	C_18_H_30_O_2_	261, 235, 54
55	37.28	Linoleic acid methyl ester	293	294	Unsaturated fatty acid ester	C_19_H_34_O_2_	66, 278
56	38.28	Arachidic acid	311	312	Saturated fatty acid	C_20_H_40_O_2_	293, 267, 59
57	38.32	Behenic acid	339	340	Saturated fatty acid	C_22_H_44_O_2_	321, 295, 59

^a^M−H is the quasi-molecular ion that results from ionization of metabolites using Electrospray Ionization technique (ESI).

^b^MSn fragments are the fragments obtained from MS2 fragmentation of ionized metabolites in collision cell of the triple quadrapole mass analyzer.

### Fishing of inflammatory target proteins for *L. camara* metabolites and networks construction

Interactions between *L. camara* endogenous metabolites with the proteins involved in inflammation were unveiled via constructing a constituent-target (C-T) network (Supplementary Fig. S2). Out of the identified 57 compounds from UPLC-MS/MS analysis, only 39 compounds were potential candidates for inflammation-related protein targets, and 35 inflammation-related target genes were eventually fished out based on screening results from STITCH public database. Regarding STITCH 5.0 database, “combined score” is the parameter utilized to evaluate the strength of interactions between the input compound and the genes. Compounds possessing high combined scores have accurate and strong interactions with their corresponding genes^13^. In this study, only compounds having interaction scores higher than 0.4 were retained^13^ (Table 2).

**Table 2:** Potential protein targets of *L. camara* constituents.

Target protein short name	Full name of protein	Interacting compound (s) (combined interaction score)
APP	Amyloid-beta precursor protein	Myricetin (1), ferulic acid (0.79), isoferulic acid (0.67), chlorogenic acid (0.83), pectolinarigenin (0.53)
BCL2	Apoptosis regulator Bcl-2	Catechin gallate (1)
BRAF	Serine/threonine-protein kinase B-raf	Myricetin (1)
CD81	CD81 antigen	Lantoic acid (0.49), pomolic acid (0.57), 3β,22β)-form, 3-O-(3-methyl-2-butenoyl) (0.64), dihydrorehmannic acid (0.65), lantadene C (0.47), 3,24-dihydroxy-12-ursen-28-oic acid; 3 β form, 3-ketone (0.42), lantanolic acid (0.43), pomonic acid (0.4), icterogenin (0.45), lantanillic acid (0.42), camaric acid (0.41), lantacin (0.51), 3,24-dihydroxy-12-oleanen-28-oic acid; 3α form, 3-ketone, 24-aldehyde (0.49), 3,22-dihydroxy-12-oleanen-28-oic acid; (3 β,22 β)-form, 3-ketone, 22-angeloyl (0.47), 3,22-dihydroxy-12-oleanen-28-oic acid; (3 β, 22 β)-form, 3-ketone, 22-(3-methyl-2-butenoyl) (0.49)
CREB1	Cyclic AMP-responsive element-binding protein 1	Pectolinarin (0.42), linaroside (0.47), pectolinarigenin (0.79), penduletin (0.4)
CXCL12	Stromal cell-derived factor 1	Ferulic acid (0.69), isoferulic acid (0.53)
FLT3	Receptor-type tyrosine-protein kinase FLT3	Myricetin (1)
FOS	Proto-oncogene c-Fos	Ferulic acid (0.74), isoferulic acid (0.56)
IL2	Interleukin-2	Scoparin (0.5), pectolinarin (0.47), narcissin (0.4), 8-epiloganin (0.42), chrysoeriol-7-O-GLUCOSIDE (0.81), linaroside (0.6), rhamnocitrin-O-GLUCOSIDE (0.57)
INSR	Insulin receptor	Myricetin (1)
JUN	Transcription factor AP-1	Ferulic acid (0.7)
KIT	Mast/stem cell growth factor receptor Kit	Pectolinarigenin (1)
LPAR1	Lysophosphatidic acid receptor 1	Linoleic acid methyl ester (0.48), Linolenic acid (0.43), myristoleic acid (0.47)
LPAR2	Lysophosphatidic acid receptor 2	Arachidic acid (0.4), behenic acid (0.4), linoleic acid methyl ester (0.48), Linolenic acid (0.43), myristoleic acid (0.47)
LPAR3	Lysophosphatidic acid receptor 3	Arachidic acid (0.4), behenic acid (0.4), linoleic acid methyl ester (0.48), Linolenic acid (0.43), myristoleic acid (0.47)
LPAR4	Lysophosphatidic acid receptor 4	Linoleic acid methyl ester (0.48), myristoleic acid (0.41)
MAPK14	Mitogen-activated protein kinase 14	Catechin gallate (1)
MAPT	Microtubule-associated protein tau	Ferulic acid (0.69), isoferulic acid (0.67), myricetin (1)
MET	Hepatocyte growth factor receptor	Catechin gallate (1)
MMP9	Matrix metalloproteinase-9	Ferulic acid (0.74), isoferulic acid (0.64)
PGF	Placenta growth factor	Afzelechin (0.59)
PIK3CG	Phosphatidylinositol 4,5-bisphosphate 3-kinase catalytic subunit gamma isoform	Myricetin (1)
PLA2G2C	Putative inactive group IIC secretory phospholipase A2	Arachidic acid (0.52), behenic acid (0.52), myristoleic acid (0.43)
PLA2G4B	Cytosolic phospholipase A2 beta	Arachidic acid (0.46), behenic acid (0.46)
PLA2G5	Calcium-dependent phospholipase A2	Arachidic acid (0.43), behenic acid (0.43)
PPARA	Peroxisome proliferator-activated receptor alpha	Arachidic acid (1), behenic acid (1)
PRKCA	Protein kinase c alpha	Linoleic acid methyl ester (0.51), verbascoside (1)
PTGER2	Prostaglandin E2 receptor EP2 subtype	Arachidic acid (0.45), behenic acid (0.45), ferulic acid (0.44), isoferulic acid (0.44)
PTPN1	Tyrosine-protein phosphatase non-receptor type 1	3,22-Dihydroxy-12-oleanen-28-oic acid; 3 β,22 β form, 3-ketone, 22-(3-methyl-2-butenoyl) (0.61), 3,22-dihydroxy-12-oleanen-28-oic acid; (3 β,22 β)-form, 3-ketone, 22-angeloyl (0.61), 3,24-dihydroxy-12-oleanen-28-oic acid; 3α;-form, 3-ketone, 24-aldehyde (0.79), 3,24-dihydroxy-12-ursen-28-oic acid; 3 β-form, 3-ketone (0.64), camaric acid (0.45), camarinic acid (0.42), dihydrorehmannic acid (0.67), icterogenin (0.54), lantadene C (0.59), lantanillic acid (0.44), lantanolic acid (0.6), lantic acid (0.61), lantoic acid (0.49), pomolic acid (1), pomonic acid (0.82), Ursoxy acid (0.58)
PTPN6	Tyrosine-protein phosphatase non-receptor type 6	3,22-Dihydroxy-12-oleanen-28-oic acid; (3β,22β)-form, 3-O-(3-methyl-2-butenoyl) (0.51), 3,24-dihydroxy-12-oleanen-28-oic acid; 3α;-form, 3-Ketone, 24-aldehyde (0.53), 3,24-dihydroxy-12-ursen-28-oic acid; 3β-form, 3-ketone (0.45), dihydrorehmannic acid (0.54), lantadene C (0.42), lantanolic acid (0.54), pomolic acid (0.54)
RELA	Transcription factor p65	Ferulic acid (0.5), isoferulic acid (0.54)
RPS6KA3	Ribosomal protein S6 kinase alpha-3	Narcissin (0.62), rhamnocitrin-O-glucoside (0.72)
SYK	Tyrosine-protein kinase SYK	Myricetin (1)
TLR2	Toll-like receptor 2	Arachidic acid (1), behenic acid (1), linoleic acid methyl ester (0.46), linolenic acid (0.48)
VEGFA	Vascular endothelial growth factor a	Afzelechin (1)

**Figure 2:**
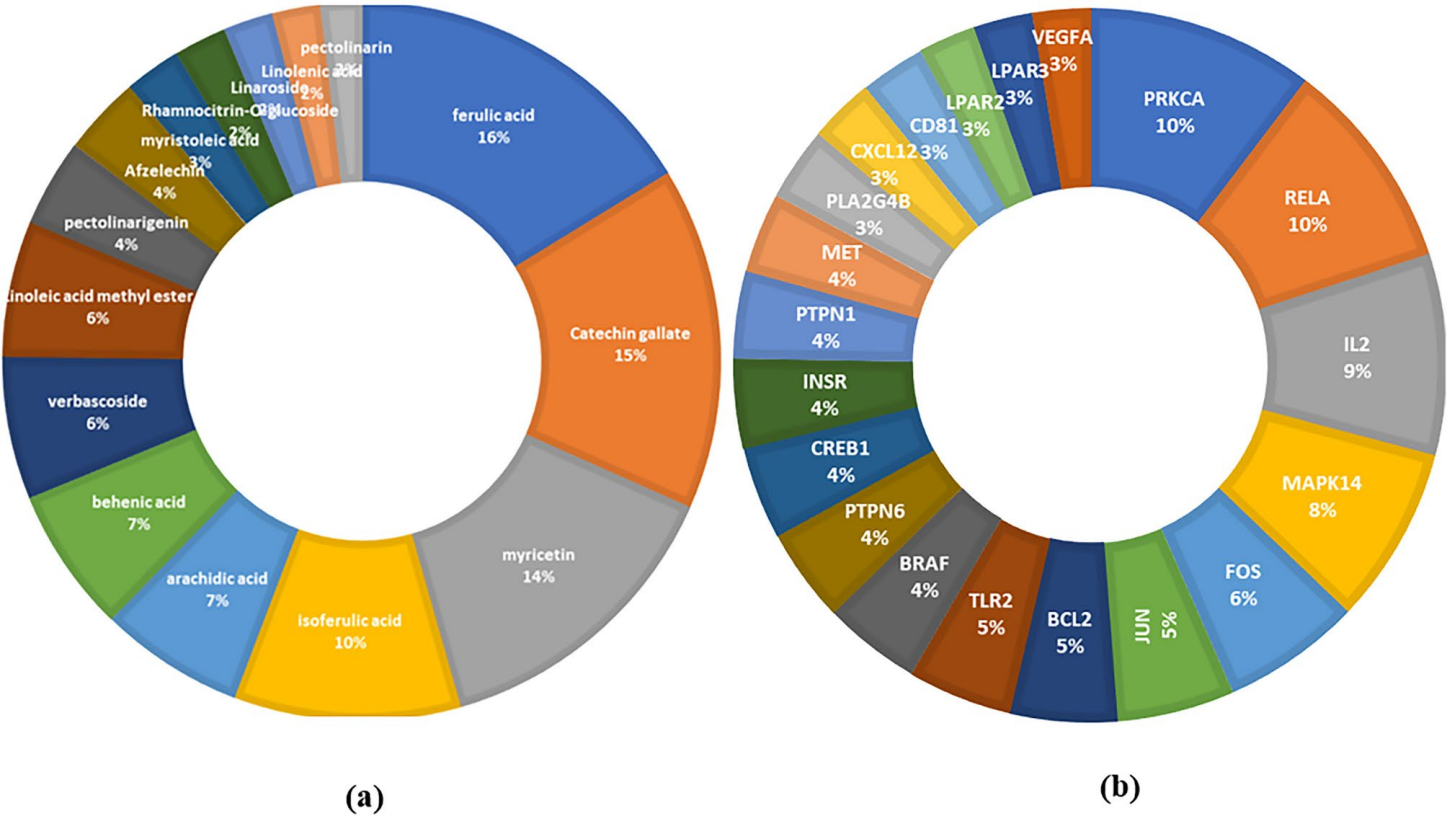
Doughnut charts showing the distributions % of the compound–target gene (C–T) interactions on *L. camara* constituents (**a**) and the identified inflammation-related proteins (**b**).

The constructed C-T network (Supplementary Fig. S2) comprised 74 nodes (39 constituents and 35 target genes) and 479 edges with an average of 3.043 targets for each constituent, indicating the multi-target properties of the *L. camara* phytoconstituents. As deduced from Fig. 2a, the highest percentages of interactions were demonstrated by ferulic acid, followed by catechin gallate, then myricetin and iso-ferulic acid. Inspection of the targeted genes (Fig. 2b, Table 2) indicated that the genes PRKCA, RELA, IL2, MAPK14 and FOS were the most enriched ones possessing the highest combined scores and interaction percentages with the constituents in the C-T network, proposing their possible key role in suppressing inflammation. In addition, protein–protein interactions were examined using STRING database then visualized through P-P network analysis. From this network, strong correlations between the identified potential anti-inflammatory target proteins were spotted suggesting that they probably regulate the functions of each other (Supplementary Fig. S3).

**Figure 3:**
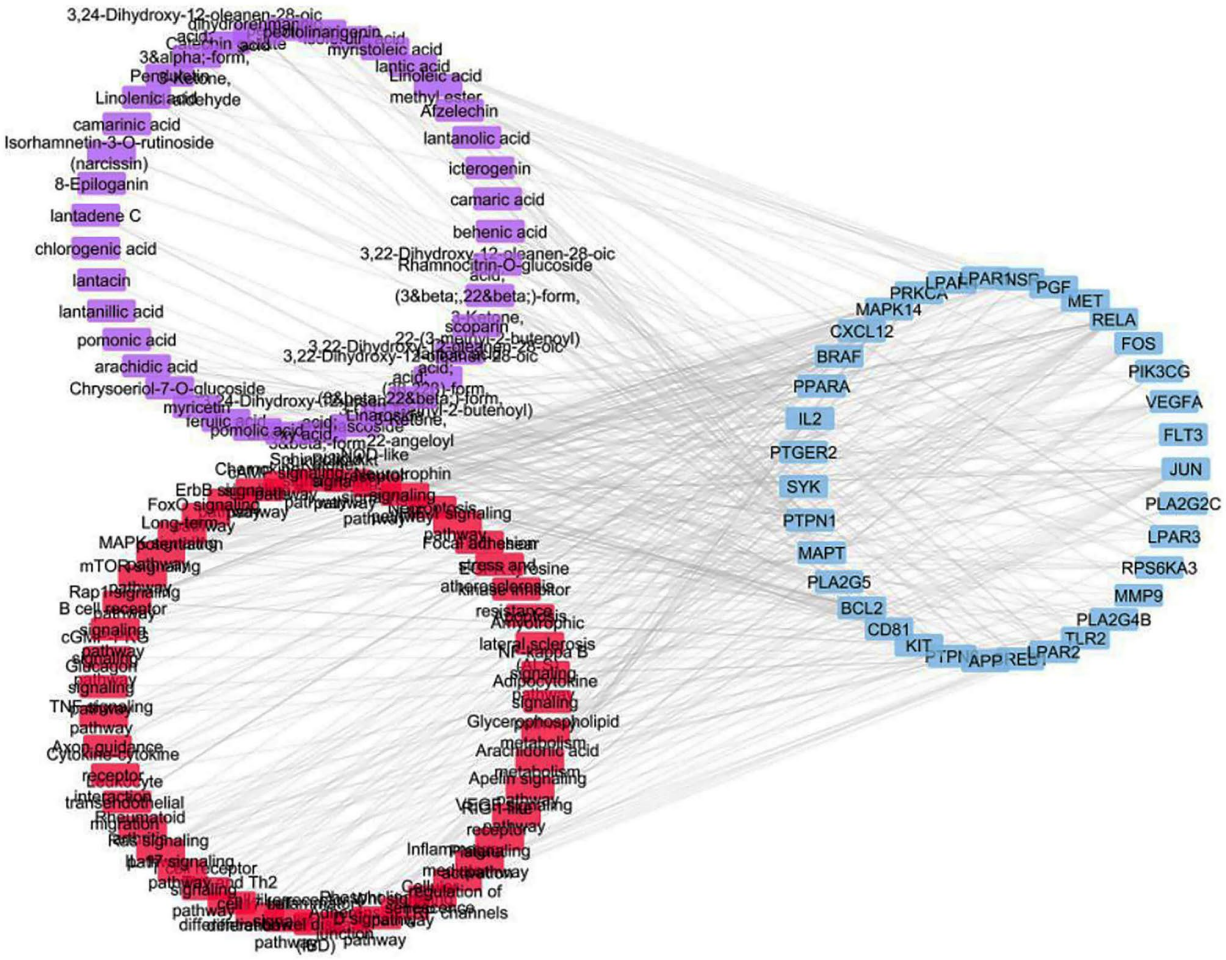
Compound–target–pathway network (compounds are represented in violet color, targets are presented in blue color and pathways are presented in red color).

Potential metabolic pathways of inflammation were explored by forwarding the target genes to the Kyoto Encyclopedia of Genes and Genomes (KEGG) pathway analysis^21–23^ where annotation was restricted to *Homo sapiens.* As shown in Supplementary Fig. S4 and Table 3, the target genes were involved in 47 inflammation-related pathways (having P-values < 0.05). The most enriched pathways were observed to be PI3K-Akt signaling pathway exhibiting the largest number of gene count followed by MAPK signaling pathway, Rap1 signaling pathway, Ras signaling pathway and Phospholipase D signaling pathway. The constructed networks were merged to generate the compound–target–pathway network (Fig. 3) which implied strong co-relations between the studied compounds and inflammation-related targets and pathways.

**Table 3:** KEGG pathway analysis of potential target genes functions.

Pathway ID	Pathway name	Gene count	False discovery rate (P-value)	Matching proteins in the network
hsa04151	PI3K-Akt signaling pathway	17	6.54E−11	IL2, FLT3, KIT, INSR, MET, PIK3CG, LPAR1, SYK, BCL2, RELA, CREB1, LPAR3, LPAR4, PRKCA, LPAR2, PGF, VEGFA
hsa04010	MAPK signaling pathway	16	1.59E−11	MAPK14, FLT3, FGF2, KIT, BRAF, INSR, FOS, MET, MAPT, JUN, RPS6KA3, RELA, PLA2G4B, PRKCA, PGF, VEGFA
hsa04015	Rap1 signaling pathway	12	9.69E−12	MAPK14, KIT, BRAF, INSR, MET, LPAR1, LPAR3, LPAR4, PRKCA, LPAR2, PGF, VEGFA
hsa04014	Ras signaling pathway	11	1.63E−09	FLT3, PLA2G2C, KIT, INSR, MET, PLA2G5, RELA, PLA2G4B, PRKCA, PGF, VEGFA
hsa04072	Phospholipase D signaling pathway	10	6.41E−08	KIT, INSR, PIK3CG, LPAR1, SYK, LPAR3, PLA2G4B, LPAR4, PRKCA, LPAR2
hsa05418	Fluid shear stress and atherosclerosis	7	4.56E−10	MAPK14, FOS, JUN, MMP9, BCL2, RELA, VEGFA
hsa04024	cAMP signaling pathway	7	2.50E−05	PTGER2, BRAF, FOS, JUN, RELA, PPARA, CREB1
hsa04510	Focal adhesion	7	0.00011	BRAF, MET, JUN, BCL2, PRKCA, PGF, VEGFA
hsa04662	B cell receptor signaling pathway	6	3.60E−08	CD81, FOS, JUN, SYK, RELA, PTPN6
hsa04668	TNF signaling pathway	6	0.00000111	MAPK14, FOS, JUN, MMP9, RELA, CREB1
hsa04660	T cell receptor signaling pathway	6	3.63E−06	IL2, MAPK14, FOS, JUN, RELA, PTPN6
hsa04722	Neurotrophin signaling pathway	6	6.06E−05	MAPK14, BRAF, JUN, RPS6KA3, BCL2, RELA
hsa04060	Cytokine-cytokine receptor interaction	6	0.0085	IL2, FLT3, KIT, MET, CXCL12, VEGFA
hsa04064	NF-kappa B signaling pathway	5	3.35E−07	SYK, CXCL12, BCL2, RELA
hsa04066	HIF-1 signaling pathway	5	3.44E−06	INSR, BCL2, RELA, PRKCA, VEGFA
hsa04620	Toll-like receptor signaling pathway	5	4.45E−06	MAPK14, TLR2, FOS, JUN, RELA
hsa01521	EGFR tyrosine kinase inhibitor resistance	5	4.51E−06	BRAF, MET, BCL2, PRKCA, VEGFA
hsa04657	IL-17 signaling pathway	5	1.40E−05	MAPK14, FOS, JUN, MMP9, RELA
hsa05323	Rheumatoid arthritis	5	4.85E−05	TLR2, FOS, JUN, CXCL12, VEGFA
hsa04659	Th17 cell differentiation	5	0.00013	IL2, MAPK14, FOS, JUN, RELA
hsa04658	Th1 and Th2 cell differentiation	5	0.00032	IL2, MAPK14, FOS, JUN, RELA
hsa04071	Sphingolipid signaling pathway	4	1.33E−13	MAPK14, BCL2, RELA, PRKCA
hsa04670	Leukocyte transendothelial migration	4	1.46E−06	MAPK14, MMP9, CXCL12, PRKCA
hsa04611	Platelet activation	4	0.00000329	MAPK14, PIK3CG, SYK, PLA2G4B
hsa04210	Apoptosis	4	6.21E−06	FOS, JUN, BCL2, RELA
hsa04750	Inflammatory mediator regulation of TRP channels	4	0.000014	MAPK14, PTGER2, PLA2G4B, PRKCA
hsa04370	VEGF signaling pathway	4	0.0000473	MAPK14, PLA2G4B, PRKCA, VEGFA
hsa05321	Inflammatory bowel disease (IBD)	4	6.06E−05	IL2, TLR2, JUN, RELA
hsa04621	NOD-like receptor signaling pathway	4	0.00013	MAPK14, JUN, BCL2, RELA
hsa04150	mTOR signaling pathway	4	0.00026	BRAF, INSR, RPS6KA3, PRKCA
hsa04520	Adherens junction	4	0.0037	INSR, MET, PTPN1, PTPN6
hsa00590	Arachidonic acid metabolism	3	8.61E−11	PLA2G2C, CBR1, PLA2G5, PLA2G4B
hsa04062	Chemokine signaling pathway	3	6.06E−05	BRAF, CXCL12, RELA
hsa04022	cGMP-PKG signaling pathway	3	0.00042	INSR, PIK3CG, CREB1
hsa04720	Long-term potentiation	3	0.00042	BRAF, RPS6KA3, PRKCA
hsa04012	ErbB signaling pathway	3	0.0014	BRAF, JUN, PRKCA
hsa04360	Axon guidance	3	0.0027	MET, CXCL12, PRKCA
hsa00564	Glycerophospholipid metabolism	3	0.0117	PLA2G2C, PLA2G5, PLA2G4B
hsa04068	FoxO signaling pathway	3	0.032	MAPK14, BRAF, INSR
hsa04920	Adipocytokine signaling pathway	2	0.0033	RELA, PPARA
hsa04218	Cellular senescence	2	0.0056	MAPK14, RELA
hsa04622	RIG-I-like receptor signaling pathway	2	0.0185	MAPK14, RELA
hsa04217	Necroptosis	2	0.0193	BCL2, PLA2G4B
hsa05014	Amyotrophic lateral sclerosis (ALS)	2	0.036	MAPK14, BCL2
hsa04310	Wnt signaling pathway	2	0.0442	JUN, PRKCA
hsa04922	Glucagon signaling pathway	2	0.0489	PPARA, CREB1
hsa04371	Apelin signaling pathway	1	0.0027	PIK3CG

### Gene ontology (GO) enrichment analysis for targets

**Figure 4:**
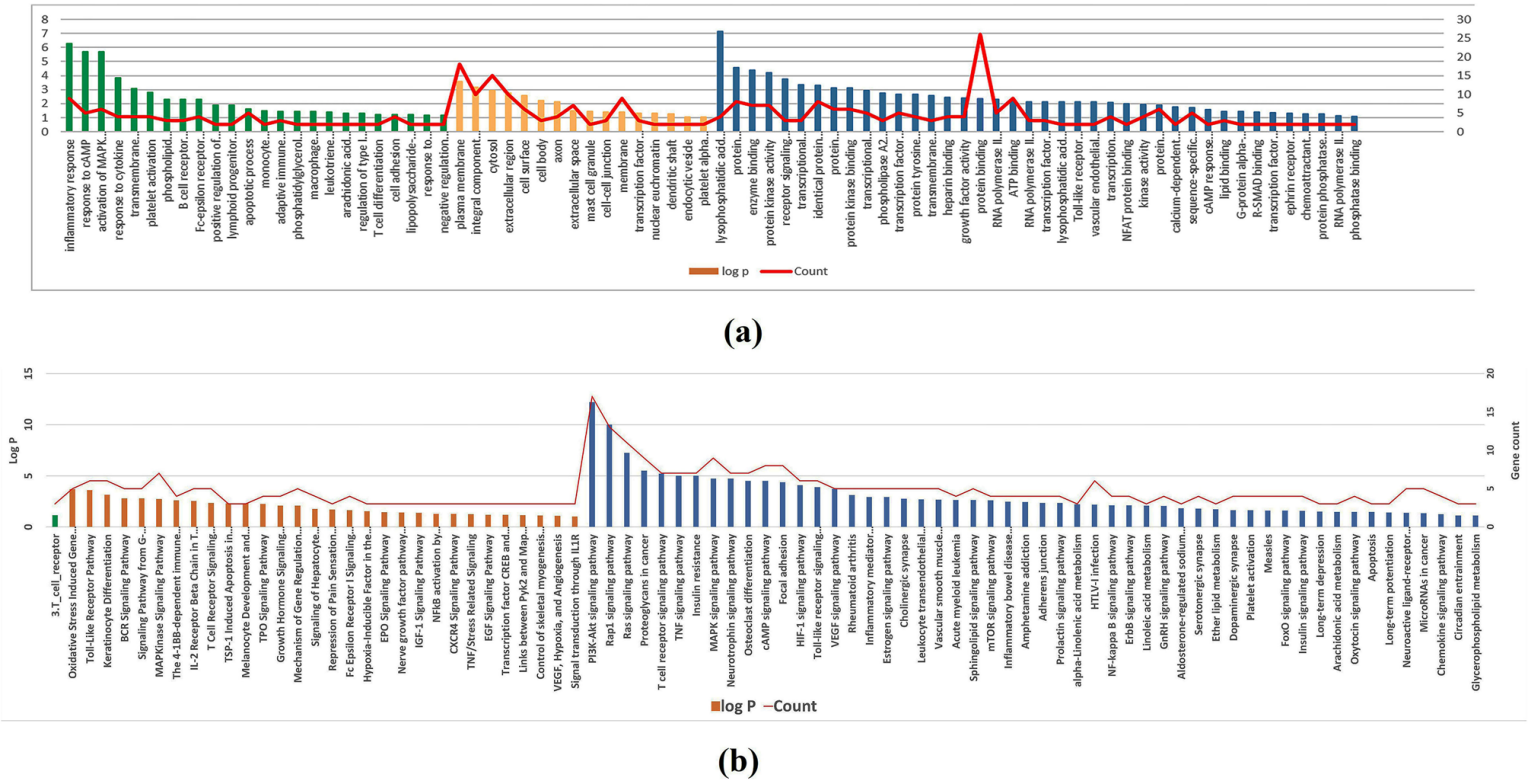
(**a**) Gene Ontology analysis of inflammation targets determined by DAVID database. Biological processes, molecular functions and cellular components are represented by green, orange and blue bars, respectively. (**b**) Major BBID (green), BIOCARTA (orange) and KEGG (blue) pathways clusters generated from DAVID database. The significance of enrichment is indicated by log P-value with bar charts. Red lines represent the number of genes enriched by each term.

Gene ontology (GO) enrichment analysis was carried out to the identified targets via importing to DAVID bioinformatics resources with limiting annotations to *Homo sapiens*, thus revealing the most enriched pathways and GO terms which have the highest log P value and gene counts. As depicted in Fig. 4a, the identified targets are associated with numerous biological processes, the most enriched ones are inflammatory response, response to cAMP, activation of MAPK activity and response to cytokine. The most significant molecular cellular components were plasma membrane, integral component of plasma membrane, cytosol and extracellular region. It was also concluded that the most enriched molecular functions were lysophosphatidic acid receptor activity, protein heterodimerization activity, enzyme binding and protein kinase activity. Nevertheless, functional annotations using DAVID bioinformatics resources revealed 1 BBID pathway named 3.T-cell receptor, and 30 BIOCARTA pathways such as: oxidative stress induced gene expression via Nrf2, Toll like receptor pathway, keratinocyte differentiation and BCR signaling pathway. Additionally, 52 KEGG pathways involving PI3K-Akt signaling pathway, Rap1 signaling pathway, Ras signaling pathway and proteoglycans in cancer were identified (Fig. 4b). All these recognized pathways possessed P-value less than or equal to 0.05, implying their striking association with inflammation.

### Molecular docking studies of *L. camara* hit compounds in the active sites of the most enriched inflammation-related target genes

The calculation of the docking XP G scores of *L. camara* top hit compounds; ferulic acid, catechin gallate, myricetin and iso-ferulic acid, against the active sites of the most enriched inflammation-related target genes; PRKCA, RELA, IL2, MAPK14 and FOS, was carried out using Glide module embedded in Schrodinger suite software. From Supplementary Table S1, it can be concluded that catechin gallate had the lowest binding energy against PRKCA, RELA and IL2, while myricetin possessed the most stable interaction against MAPK14 and FOS.

### Validation of molecular docking protocol

Validation procedures for each docking software were attained using two methods. First is the redocking procedure which evaluates the accuracy of the docking poses and during which, the co-crystallized ligands were docked back into the receptor binding cavity. The re-docked complex was superimposed on to the reference co-crystallized complex and the RMSD value between the initial conformation and the re-docked one is calculated. A cut off value of 2 Å was set; therefore, complexes encompassing above this value were considered incorrect^24^. For each of the studied proteins: 4RA4, 1M49 and 6HWU, the re-docked complex was superimposed on to the reference co-crystallized complex to a great extent (Supplementary Fig. S5). Moreover, the RMSD value between the initial conformation and the re-docked one was calculated and all the three crystallographic structures displayed good values of 1.172, 0.386, 0.558 respectively (supplementary Table S2) indicating the efficiency of the docking protocol.

Second is the utilization of enrichment calculations that are crucial for evaluating the quality of scoring and eliminating random or by chance selection of actives^25^. A validation set comprising active compounds for each of the investigated proteins was seeded into 1000 built-in Schrodinger^®^ decoys. Decoys are compounds that are similar in physical properties with respect to the reference ligand that might not bind effectively to a protein^26^. Validation parameters such as receiver operating characteristic (ROC), AUC-ROC, BEDROC and enrichment factor (EF at 2%, 5% and 10%) were then estimated. From the ROC plots, the area under the curve (AUC) computed the probability of how highly a randomly selected active is ranked compared to a randomly chosen decoy. The ideal range of AUC is 0–1, a value near ≤ 0.5 indicates that the software randomly selects true actives and false actives, where a value close to 1 highlights greater possibility to identify true actives before false ones^25^. As depicted in (supplementary Table S2), it was observed that all proteins scored promising AUC-ROC values. Comparing EF values revealed that the investigated proteins were able to extract actives from a seeded random set, when the top 2, 5 and 10% of the total set were considered, noting that the maximum attainable enrichment factors are 50, 20, and 10 for EF(2%), EF(5%), and EF(10%), respectively^27^. Using BEDROC as a criterion to assess early recognition of actives from decoys at different tuning parameter value *α*^28^, all the proteins recorded the high scores at all *α* values. To conclude, all the enrichment values obtained for each docking procedure suggested that GLIDE software was able to filter the enriched database efficiently.

### ADME filtration of *L. camara* top hit compounds

QikProp module was utilized to calculate the ADME characteristics of the *L. camara* hit compounds, in order to assess their drug-likeness*. L. camara* hit compounds were regarded as drug candidates as they conformed to Lipinski's rule of 5^29^, and Jorgensen’s rule of 3^30^ (Supplementary Table S3).

### In vitro cytotoxicity and anti-inflammatory activity of *L. camara* extract

**Figure 5:**
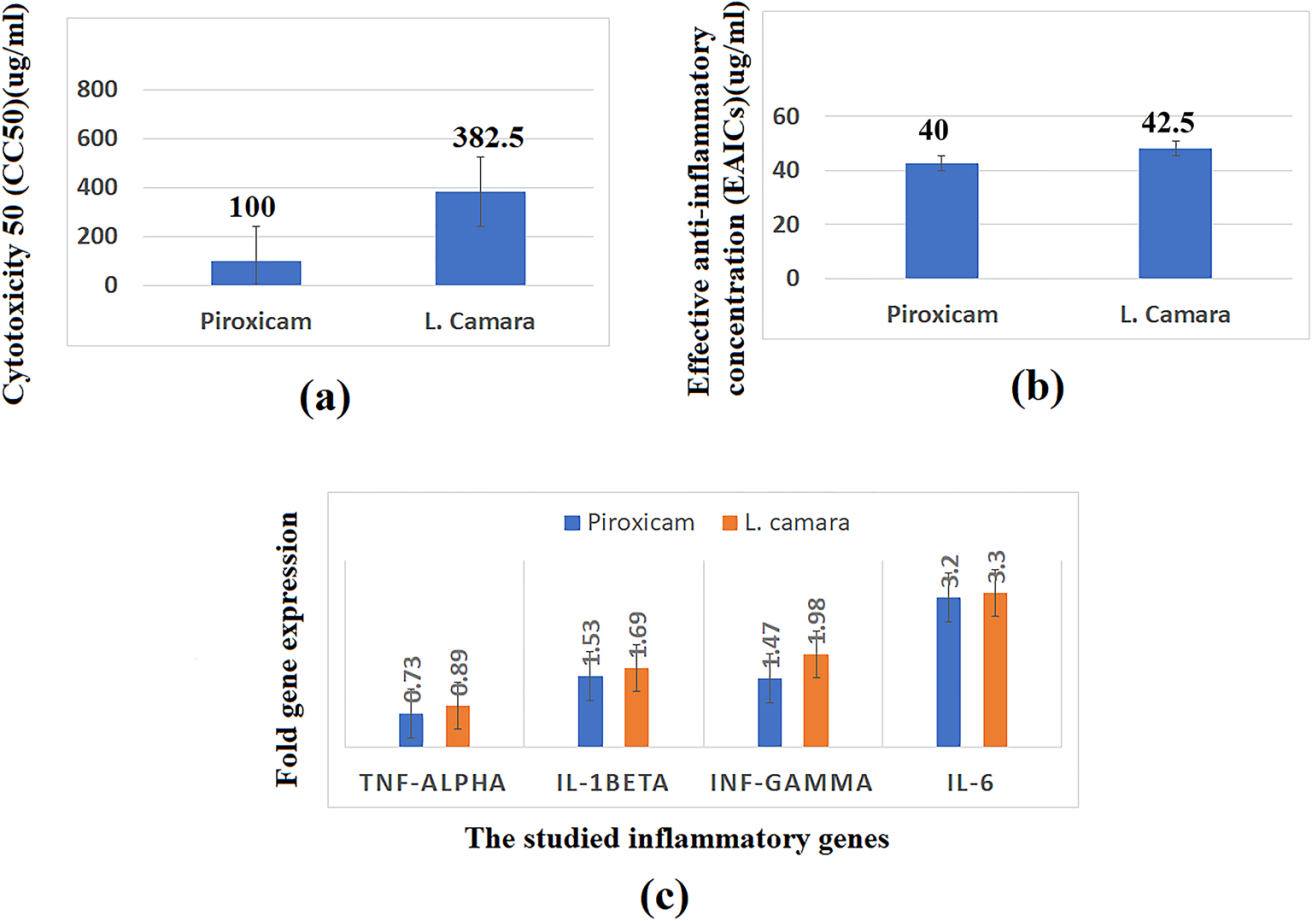
Bar chart showing (**a**) cytotoxicity (CC50 µg/mL), (**b**) effective anti-inflammatory concentrations (EAICS) (µg/mL) of *L. camara* extract and piroxicam, (**c**) TNF-*α*, IL-1*β*, INF-*γ*, IL-6 (fold change in gene expression) by *L. camara* extract and piroxicam (standard anti-inflammatory drug).

In order to assess the safety of the tested extract, the cell cytotoxicity 50 (CC50), which is the drug concentration required for reducing the cell viability by 50%, was determined for the extract and the standard anti-inflammatory drug (piroxicam) using MTT test. The tested extract showed higher CC50 value (382.5 µg/mL) than that of piroxicam (100 µg/mL) indicating that the extract is safer than piroxicam (Fig. 5a). Afterwards, anti-inflammatory activities of the extract compared to piroxicam were estimated using lipopolysaccharides (LPS)-stimulated WBC cells (Fig. 5b). Both extract and piroxicam showed comparable effective anti-inflammatory concentrations (EAICs) (48.08 µg/mL and 42.50 µg/mL, respectively), suggesting the promising activity of the extract as anti-inflammatory candidate. To determine the mechanism of anti-inflammatory activity at the genetic level, the gene expression of four pro-inflammatory markers (TNF-*α*, IL-1*β*, INF-γ, IL-6) was measured by real time polymerase chain reaction (PCR) in normal WBCs and lipopolysaccharide (LPS)-treated WBCs (Fig. 5c). Regarding TNF-*α*, lipopolysaccharide (LPS) upregulated the expression of this gene by 2.1-folds. Upon treatment of the WBCs with the tested extract this upregulation was abolished to 0.89-fold which was comparable to that exerted by piroxicam (0.73-fold). Meanwhile, LPS upregulated the expression of IL-1*β* by 5.23-folds which was attenuated by the extract to 1.69-folds. This value was in close agreement to that obtained by piroxicam (1.53-folds). Interestingly, the upregulation of the gene expression of INF-γ and IL-6 was significantly decreased by the tested extract and piroxicam to a similar level (error bars were shown in Fig. 5 and p values for all experiments were less than 0.05). It can be concluded that *L. camara* extract can serve as potential anti-inflammatory natural product assigning to its noticeable inhibition of the upregulated TNF-*α*, IL-1*β*, INF-γ, IL-6 expression levels. These results were compatible with that obtained from network pharmacology and molecular docking analyses that revealed the multi-target and multi-pathways nature of the tested extract regarding anti-inflammatory activity.

## Discussion

LC–MS/MS analysis results in 57 identified metabolites which belong to different chemical classes such as, flavonoids, phenolic acids, iridoids, phenyl ethanoid glycosides, triterpenes, and fatty acids.

### Flavonoids

This class is represented by 15 peaks (8, 9, 10, 16, 17, 18, 20, 22–29), peaks 9, 10, 16, 17, 20 and 22 represented flavonoid glycosides. Compound 10 showed daughter MS^2^ fragment of its aglycone (M-H-2CH_3_) at 299 Da^31^ thus it was identified as a di-methoxylated flavone. Based on the mass data with that reported in literature, compound 10 was identified as pectolinarin^32^. Compound 8 is a glycoside with rutin sugar part as it showed a characteristic peak at 315 Da (M-H-308) along with its characteristic daughter fragments at 300, 271 and 255 Da. By referring to literature it was tentatively identified as isorhamnetin-O-rutinoside^33^. Compounds 16 and 17 showed (M-H-162) peak that indicated the loss of one hexose unit. Compound 20 was C-glycoside that deduced from its characteristic fragments at 371, 341 and 298 Da as scoparin^34^. Compounds (23–29) represented flavone aglycones and by referring to literature they were tentatively identified as afzelechin, myrcetin, kaempferol, chrysoeriol, cirsiliol, pectolinarigenin, Penduletin and 3′,4′-Dimethoxy-7-hydroxyflavanone^35–40^. Moreover, compound 8 was identified as catechin gallate^41^.

### Phenolic acids

This class was represented by four peaks (2, 3, 4 and 5). Mass fragmentation of phenolic acids is generally characterized by loss of water and CO_2_ and loss of methyl groups in case of methylated phenolic acids^42^. By referring to literature, 2, 3, 4 and 5 were identified as gallic acid, chlorogenic acid, ferulic and iso-ferulic acid^42–45^.

### Phenylethanoid glycosides

This class was represented by two peaks (11 and 12). Phenyletanoids are *β*-glucopyranose directly attached to a hydroxyphenyl ethyl moiety. Moreover, at the positions C-4 and C-6 the substitution by hydroxyl derivatives of cinnamic acid (such as caffeoyl and feruloyl) usually occurs. At the C-2 or C-3 position of *β-*glucopyranose, another sugar moiety is usually located^46^. Two highly abundant mass fragments at 161 and 179 Da indicated the presence of caffeoyl moiety attached to the glucose unit^47^. Meanwhile, three sequential losses of caffeoyl moiety, deoxyhexose (rhamnose) moiety (M-H-162-146)^47^ and glucose unit from the parent ion followed by dehydration to yield another daughter fragment (M-H-162-146-162-18) at 135 Da assigned for anhydrophenolethanol moiety^48^. Compounds 11 and 12 were tentatively identified as verbascoside^48^ and isoverbascoside^49^, respectively.

### Iridoid glycosides

Five peaks represented this class (13, 14, 15, 19 and 30). Generally, they showed the characteristic peak (M+HCOO-H)^−^ and they showed their major MS2 fragment (M−H)^−^. Formate anion (M+HCOO-H)^−^ is commonly resulted from iridoid glycosides bearing an ester group or a carboxyl group at C-4^50^. Compound 13 showed characteristic daughter peaks at 371 Da, 345 Da and 209 Da owing to loss of water, CO_2_ and glucose moieties, respectively^51^. By referring to literature it was tentatively identified as theveside. Moreover, compound 15 showed characteristic peaks as a result of losing methoxy group (M−H-30) at 359 Da. Furthermore, there were fragments due to loss of glucose unit (M−H-162) from the precursor ion at 259 Da along with a methyl ester loss represented by the mass fragment (M−H-60) at 329 Da with subsequent dehydration to afford the major product ion (M−H-78) at 311 Da, respectively. Based on the mentioned information and by referring to literature, compounds 14, 15 and 19 were tentatively identified as geniposide^52^, 8-epiloganin^53,54^ and durantoside I, respectively^55^. Moreover, peak 30 was tentatively identified as lamiide^56^.

### Triterpenes

Oleanane-type triterpenes were represented by 12 peaks (peaks from 31 to 42) and ursane-type triterpenes were represented by 10 peaks (peaks from 43 to 52). Oleanane and ursane-type triterpenes were characterized by the presence of the most important mass fragments due to loss of angeloyl or methyl butenoyl or hydroxyl moiety followed by loss of CO_2_. Based on this information and by referring to literature, compounds from 31 to 42 were tentatively identified as 3,12,13-trihydroxy-28-oleananoic acid; 3-ketone, 3,24-dioxo-12-oleanen-28-oic acid, 24-hydroxy-3-oxo-12-oleanen-28-oic acid, lantanolic acid, icterogenin, lantanilic acid, camaric acid, 22-tigloyloxylantanolic acid, lantadene A, lantadene B and dihydrorehmannic acid, respectively^57–60^. On the other hand, compounds from 43 to 52 were tentatively identified as, lantoic acid, 3,24-dioxo-12-ursen-28-oic acid, 24-hydroxy-3-oxo-12-ursen-28-oic acid, 3,25-epoxy-3-hydroxy-12-ursen-28-oic acid, pomonic acid, lantic acid, pomolic acid, camarinic acid, ursoxy acid and lantacin, respectively^57–60^.

### Fatty acids

Three peaks (53, 54 and 55) represented unsaturated fatty acids. The mass fragmentation of unsaturated fatty acids is represented by two characteristic fragments due to loss of water and CO_2_^61^ along with their characteristic fragment at 54 m/z that result from double-bond transfer and α-cleavage^62,63^. Compounds 53, 54 and 55 were identified as myristoleic acid, linolenic acid and linoleic acid methyl ester, respectively^64^. Meanwhile, two peaks (56 and 57) represented saturated fatty acids, the mass fragmentation of saturated fatty acids is represented by two characteristic fragments result from loss of water and CO_2_^65^ along with the fragment of Mclafferty rearrangement that was detected at 59 Da^66^. Compounds 56 and 57 were tentatively identified as arachidic acid and behenic acid, respectively^64^.

PubMed literature review was implemented to validate the role of the hit compounds identified from network pharmacology analysis in alleviation of inflammation. As can be observed in Supplementary Table S4, ferulic acid precluded methotrexate-induced hepatotoxicity via inducing Nrf2/HO-1 signaling and PPARγ, as well as abolishing oxidative stress and inflammation^67^. Catechin gallate diminished the levels of cyclo-oxygenase and lipoxygenase inflammatory mediators thus alleviated UV radiation-induced erythema^68^. Another previous work confirmed the protective effect of myricetin against liver fibrosis in a diet-induced non-alcoholic steatohepatitis rat model through inhibiting the TREM-1-TLR2/4-MyD88 signaling molecules in macrophages^69^. Meanwhile, isoferulic acid attenuated the production of PI3K/Akt-dependent NF-κB activity, thus, could serve as a potential drug for treating neuritis and other neuronal ailments^70^.

In addition, several studies have documented the relation between the recognized most enriched genes and inflammation. For example, controlling the expression of PRKCA levels relieved Barrett's esophagus, esophagitis^71^, multiple sclerosis^72^ and inhibited LPS-induced acute lung injury and inflammatory response^73^. Moreover, regulation of nuclear-cytoplasmic shuttling of RELA aids in attenuation of inflammation^74^. It was also proved that loss of epithelial RELA results in deregulated intestinal proliferative/apoptotic homeostasis and susceptibility to inflammation^75^. Furthermore, expression and induction of a pancreatitis-associated protein (PAP1) depended on RelA/p65 levels, suggesting its multidimensional roles in treating cerulein pancreatitis^76^. Also, allergic inflammation was claimed to be influenced by nuclear factor κB1/RelA expression in human lung epithelial cells^77^. Meanwhile, interleukin-2 (IL-2) is the canonical T-cell growth factor that stimulates clonal expansion of T cells following antigen stimulation, hence plays a critical role in orchestrating optimal immune and inflammatory responses^78^. Therefore, targeting such protein contributes to alleviate inflammatory bowel diseases as well as suppressing inflammation synergized by respiratory viral infections^79^. Additionally, P38α/MAPK14 is intracellular signaling regulator involved in biosynthesis of inflammatory mediator cytokines as TNF-*α*, IL-1, IL-6, and IL-1*β*, which induced the production of inflammatory proteins such as iNOS, NF-kB, and COX-2^80^. Also, regulation of MAPK14 expression prevented aggravation of myocarditis^81^, multiple sclerosis^82^ and inflammatory bowel diseases^83^. Other recent work confirmed the vital role of MAPK14 in relieving the inhibitory control by autophagy on inflammation in response to a stress signal^84^.

**Figure 6:**
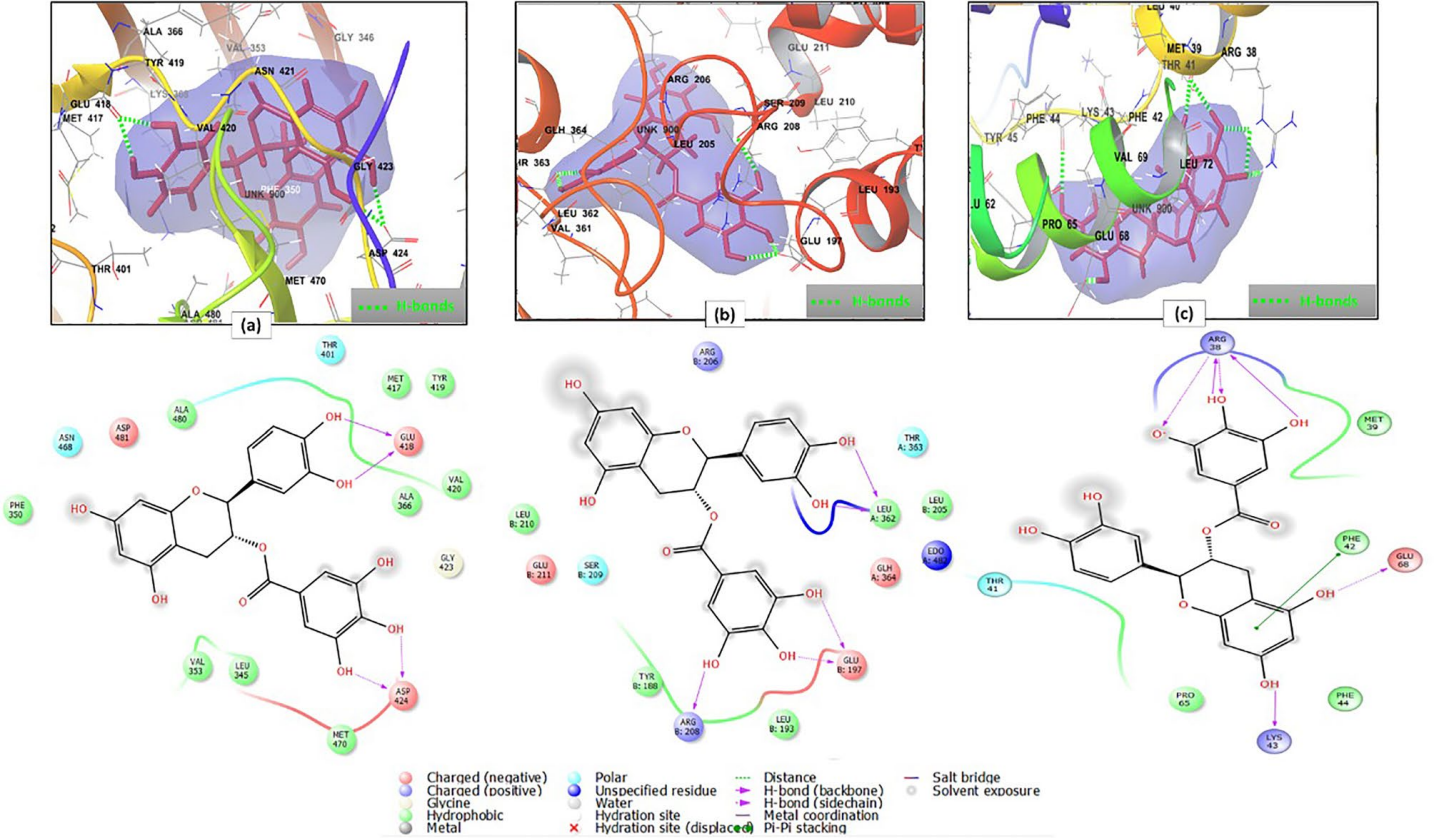
2D and 3D interaction diagrams of (**a**) catechin gallate in the active site of protein kinase C alpha type (PDB ID 4RA4) (**b**) catechin gallate in the active site of transcription factor p65 (PDB ID 3QXY) (**c**) catechin gallate in the active site of interleukin-2 (PDB ID 1M49).

Molecular docking analysis revealed the strong binding of the top hit compounds on the active sites of the most enriched genes. For example; the 2D and 3D interaction diagrams of catechin gallate in the active site of protein kinase C alpha type (PBD ID 4RA4) (Fig. 6a) showed that the strong binding—as expressed by its XP G score- was attributed to the formation of two hydrogen bonds between 3 and 4′ hydroxyl groups and Glu418, two hydrogen bonds between 3 and 4″ hydroxyl groups and Asp424, In addition to hydrophobic interactions with Phe350, Ala480, Met417, Tyr419, Ala366, Val420, Met470, Leu345 and Val353. Moreover, polar interactions with Asn468 and Thr401 and charged negative interactions with Asp481, Glu418 and Asp424 were observed^85^ (Supplementary Table S5).

Meanwhile, catechin gallate occupied the active site of transcription factor p65 (PBD ID 3QXY) with two hydrogen bonds between 3 and 4′ hydroxyl groups and LeuA 362, two hydrogen bonds between 3″, 4″ hydroxyl groups and GluB 197 and another one between 5″ hydroxyl group and ArgB 208. Additionally, hydrophobic interactions with LeuB 205, LeuA 362, LeuB 193, TyrB 188 and LeuB 210, and polar interactions with ThrA 363 and SerB 209 were denoted. Moreover, charged positive interactions with ArgB 206 and ArgB 208, and charged negative interactions with GlhA 364, GluB 197 and GluB 211 were involved in binding^82^ (Fig. 6b, Supplementary Table S5).

However, binding of catechin gallate with interleukin-2 (PBD ID 1M49) showed two hydrogen bonds between the hydroxyl groups at C-5 and C-7 and Glu68 and Lys43, respectively, together with four hydrogen bonds between 3″, 4″ and 5″ hydroxyl groups and Arg38. Also, a pi–pi stacking interaction between the aromatic ring A of the flavone moiety and Phe42 and hydrophobic interactions with Pro65, Phe44, Phe42 and Met39 were unveiled. Two charged positive interactions with Lys43 and Arg38, one charged negative interaction with Glu 68, beside one polar interaction with Thr41 were also deduced^86^ (Fig. 6c, Supplementary Table S5).

**Figure 7:**
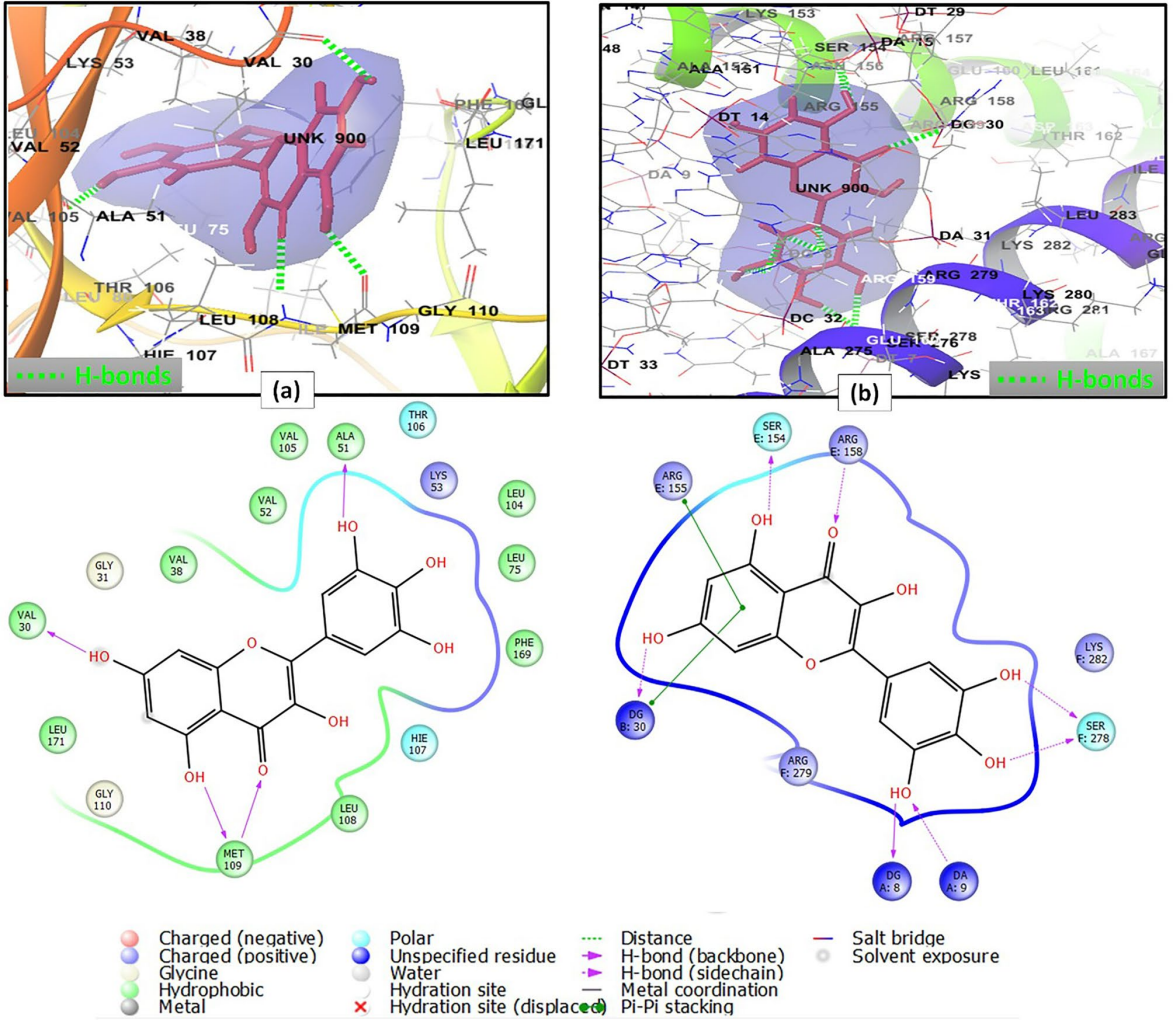
2D and 3D interaction diagrams of (**a**) myricetin in the active site of mitogen-activated protein kinase 14 (PDB ID 6HWU) (**b**) myricetin in the active site of proto-oncogene c-Fos (PDB ID 1FOS).

On the other hand, the interaction pattern of myricetin with mitogen-activated protein kinase 14 (PBD ID 6HWU) included the formation of four hydrogen bonds between the following pairs: 3′ hydroxyl group and Ala51; 4 carbonyl group, 5 hydroxyl group and Met109; and 7 hydroxyl group and Val30; in addition to hydrophobic interactions with Val30, Leu171, Met109, Leu108, Val105, Ala51, Val52, Leu104, Leu75, Phe169 and Val38. There were also polar interactions with Thr106 and Hie107 and a charged positive interaction with Lys53^87^ (Fig. 7a, Supplementary Table S5). Furthermore, the binding mode of myricetin with proto-oncogene c-Fos (PBD ID 1FOS) revealed the presence of four hydrogen bonds between 4 carbonyl group and ArgE 158, C-5 hydroxyl group and SerE 154, C-3′, C-4′ hydroxyl groups and SerF 278. Myricetin also engaged in a pi–pi stacking interaction through its flavone aromatic ring A moiety and ArgE 155. Charged positive interactions with the backbone amino acid residues ArgE 155, ArgE 158, LysF 282, ArgF 279, polar interactions with SerE 154 and SerF 278, and a hydrophobic interaction with AlaE 151 were also considered^88^ (Fig. 7b, Supplementary Table S5).

The drug-likeness of compounds can be predicted by applying Lipinski's rule of 5. As claimed by Lipinski's rule of 5, a compound of known pharmacological activity is regarded active (having good absorption and/or permeation) if it possesses less than 10 hydrogen-bond acceptors (acptHB), less than 5 hydrogen-bond donors (donorHB), a molecular weight (mol_MW) lower than 500 Da and a calculated QPlogPo/wvalue less than five^29^. Only compounds conformed to minimally three of the above characteristics were considered active.

In addition, the oral bioavailability (OB) of the top hit phytoconstituents was evaluated using the descriptor Jorgensen’s rule of 3^30^. Only compounds demonstrating OB ≥ 30% were considered active. The hit *L. camara* constituents obey the above criteria and hence, were considered as drug candidates (Supplementary Table S3).

## Methods

### Chemicals and plant material

All chemicals utilized in this study were procured from (St. Louis, Mo., USA). Dimethyl sulfoxide (DMSO), piroxicam, lipopolysaccharides (LPS), SYBR green master mix, trypan blue and MTT (3-(4,5-dimethylthiazol-2-yl)-2,5-diphenyl tetrazolium bromide) dyes, RNA and cDNA extraction kits, nuclease free water, RNase inhibitor and reverse transcriptase were bought from Sigma (St. Louis, Mo., USA). Fetal Bovine serum, Roswell Park Memorial Institute (RPMI) 1640 Medium, l-glutamine were obtained from Lonza (Belgium). dT primer, dNTPs (deoxynucleotide triphosphate) were purchased from Thermo Fisher Scientific.

The aerial parts of *L. camara* were collected from Antoniades Garden, Alexandria, Egypt with permission from the Agriculture Research Center, Giza, Egypt at "9 Cairo University Road, Giza District, Giza Governorate". The plant collection was accomplished in accordance with the national guidelines. The identity of the plant was confirmed by Dr. Therese Labib, specialist of plant identification in El Orman Garden, Cairo, Egypt. A voucher specimen (No. LC-250) was deposited at the herbarium of the Department of Pharmacognosy, Faculty of Pharmacy, Alexandria University.

### Preparation of *L. camara* extract

Air-dried powdered leaves of *L. camara* (500 g) were extracted by sonication in 1 L of 95% ethanol in an ultrasonic bath apparatus 28 kHz/1100 W (3 L Alpha Plus, Japan) for 30 min at 35 °C. The obtained extract was filtered, and the procedure was repeated twice. The obtained extracts were combined and evaporated to dryness under reduced pressure using rotary evaporator at 45 °C to obtain 200 g dry residue. A portion of the dry residue of *L. camara* extract was dissolved in HPLC-grade methanol to obtain a sample solution of concentration 1 mg/mL. This sample solution was filtered using a membrane disc filter (0.2 μm), then degassed by sonication. After that, a full loop injection volume (10 μL) of this solution was injected into the chromatographic column.

### Analysis of *L. camara* extract using UPLC-MS/MS technique

The chromatographic analysis was accomplished using an UPLC XEVO TQD triple quadruple instrument Waters Corporation, Milford, MA01757 USA equipped with a Waters Acquity QSM pump, a LC-2040 autosampler, degasser in addition to Waters Acquity CM detector. The dimensions of Waters Acquity UPLC BEH C18 column was 50 mm (length), 2.1 mm (internal diameter) and 1.7 μm (particle size). The operation of the column was at a flow rate of 0.2 mL/ min and the system was thermostated at 30 °C. The mobile phase that used for analyses consisted of two phases; phase A: ultrapure water + 0.1% formic acid, and phase B: methanol + 0.1% formic acid. Elution was gradient one and its program was as following: 0.0–2.0 min, 10% B; 2.0–5.0 min, 30% B; 5.0–15.0 min, 70% B; 22.0 min, 90% B; 22.0–25.0 min, 90% B; 26.0 min, 100% B; 26.0–29.0 min, 100% B; 30.0 min, 10% B. Then 4 min were set at the initial conditions to re-equilibrate the column.

The mass spectrometric analysis and metabolites annotation were carried out according to the method described by Darwish et al.^89^ as shown in the Supplementary data.

### Network pharmacology-based analysis

The 2D structures of the identified compounds yielded from UPLC-MS/MS analysis were converted to SMILES format using Schrodinger software (LLC, New York, NY, 2015), then furtherly subjected to network pharmacology- based analysis. The identification of the target genes linked to the selected constituents was performed using STITCH database (http://stitch.embl.de/, ver.5.0) with the ‘*Homo sapiens’* species settings. UniProt (http://www.uniprot.org/)^90,91^ was utilized for retrieving gene information including name, gene ID and accession number. To retrieve information about functional annotation and the signaling pathways, bioprocesses, cellular components and molecular functions that were highly associated with inflammation target proteins, DAVID ver. 6.8 (https://david.ncifcrf.gov/) and the Kyoto Encyclopedia of Genes and Genomes (KEGG) pathways (http://www.genome.jp/kegg/pathway.html) were employed. An adjusted p-value < 0.05 was set as a cut-off value for enriching the significance of contributing pathways to inflammation.

Three types of networks: constituent-target gene, gene-pathway, and constituent-gene-pathway networks were constructed and visualized by Cytoscape 3.8.2 (http://www.cytoscape.org/) in order to visualize the interactions between compounds, target proteins and inflammation-related pathways. In the graphical network, each constituent, gene protein and pathway were described by node, and the interactions were encoded by edges. The network parameters were calculated using the network analyzer plug-in in Cytoscape where the weight of nodes in each constructed network was evaluated using Cytoscape combined score of interactions. Protein–protein interaction network (PPI network) was constructed using STRING database (https://string-db.org/).

### Molecular docking studies

Molecular docking studies were performed using Glide module integrated in Schrodinger^®^ software. The Protein Data Bank (PDB) was utilized to retrieve the crystal structures of the most enriched target proteins recognized from network pharmacology analysis, named; protein kinase C alpha type (4RA4), transcription factor p65 (3QXY), interleukin-2 (1M49), mitogen-activated protein kinase 14 (6HWU) and proto-oncogene c-Fos (1FOS). These crystal structures were saved as pdb files for further preparation using the PrepWiz module. Location of the binding site for the docking experiments was determined using the receptor grid generation module. Some protein models have no co-crystallized ligands (ex: 3QXY and 1FOS), so the ligand was set as the centroid of specified selected residues retrieved from literature. Hence, the size of the receptor grid predetermined as (20 × 20 × 20 Å^3^) was adjusted to accommodate ligands with size ≤ 20 Å to exclude large molecules with overestimated docking scores. For other models with co-crystallized ligands (ex: 4RA4, 1M49 and 6HWU) the boxes enclosing the centroids of co-crystallized ligands were set as the grids. 3D-structures of the top hit compounds recognized from network pharmacology analysis (ferulic acid, catechin gallate, myricetin and isoferulic acid) were imported as SDF files to be prepared using Ligprep module generating molecules with correct chiralities, ionization states, tautomers, stereochemistries and ring conformations. The generated compounds from the LigPrep file were flexibily docked using extra precision (XP) docking, and 2D and 3D ligand-target interactions were visualized in maestro interface.

The docking protocol was validated using two methods: (i) redocking of the co-crystallized ligands into the binding sites of their corresponding proteins then the resulting complexes were superimposed on to the reference co-crystallized complexes and the root mean square deviation (RMSD) was calculated. This was done to ensure exact binding of the inhibitor to the active site where less deviation compared to the actual co-crystallized complex is more favorable. This method was exclusively performed for proteins bearing co-crystallized ligands (Protein kinase C alpha type, 4RA4; Interleukin-2, 1M49; and Mitogen-activated protein kinase 14, 6HWU). (ii) Enrichment calculations: for each of the investigated proteins, a validation set composed of known active ligands compiled from literature was constructed (Supplementary Table S6). The validation set compounds were seeded in 1000 Schrodinger^®^ built-in decoys then docked against the active site of target protein using XP mode. Protein–ligand complexes were validated using GLIDE enrichment calculator using numerous validation parameters such as receiver operating characteristic (ROC), AUC- ROC, BEDROC and enrichment factor (EF at 2%, 5% and 10%). These calculations aimed to enrich the docking procedure and to discriminate active compounds from non-active ones thus, avoiding false positive hits production.

### ADME and drug-likeness of top hit compounds

The top hit constituents related to inflammation were assessed for drug-likeness by calculating in-silico absorption, distribution, metabolism, and excretion (ADME) criteria and adopting Lipinski's rule of five^29^, by the aid of Qikprop module (Schrodinger suite 2017A). Only compounds with predicted oral bioavailability ≥ 30 and satisfying at least three criteria from Lipinski's rule of five were considered active.

### In vitro cytotoxicity and anti-inflammatory activity testing

It was carried out according to the method described by Darwish et al.^92^ as shown in the Supplementary data.

## Conclusion

In this study, the phytoconstituents of *L. camara* extract were identified using UPLC-MS/MS analysis, then they were subjected to network pharmacology analysis that declared ferulic acid, catechin gallate, myricetin and isoferulic acid as the endogenous metabolites mostly associated to inflammation, and PRKCA, RELA, IL2, MAPK 14 and FOS as the main inflammation-related genes. The identified target genes were involved in 47 inflammation-related pathways, where the most enriched ones were PI3K-Akt signaling and MAPK signaling pathways. Molecular docking of top hit compounds on the active sites of the most enriched genes revealed that catechin gallate possessed the lowest binding energy against PRKCA, RELA and IL2, while myricetin exhibited the most stable interaction against MAPK14 and FOS. The extract was then forwarded to in vitro cytotoxicity and anti-inflammatory testing indicating comparable results to those of piroxicam. This study provides a profound explanation of the mechanism of the proposed anti-inflammatory activity of *L. camara* and recommends this plant as a source of potential anti-inflammatory agents. Further in vivo and clinical studies are recommended to affirm our outcomes.

## Supplementary Information

Supplementary Information.

## Data Availability

All data generated or analyzed during this study are included in this article (and its supplementary information files).
