# Supplementary material for: Metabolic profiling of Lantana camara L. using UPLC-MS/MS and revealing its inflammation-related targets using network pharmacology-based and molecular docking analyses
Source: Sci Rep. 2022 Sep 1;12:14828. doi: 10.1038/s41598-022-19137-0 (PMC9436993; doi:10.1038/s41598-022-19137-0)
Supplement: Supplementary file 1 — Supplementary Information. [file 41598_2022_19137_MOESM1_ESM.pdf]

# Metabolic profiling of *Lantana camara* L. using UPLC-MS/MS and revealing its inflammation-related targets using network pharmacology-based and molecular docking analyses

Alaa A. El-Banna[1]*, Reham S. Darwish[1], Doaa A. Ghareeb[2,3,4], Abdelrahman M. Yassin[2], Shaymaa A. Abdulmalek[2,3,4], Hend M. Dawood[1]

[1] Department of Pharmacognosy, Faculty of Pharmacy, Alexandria University, Alexandria, 21521, Egypt.

[2] Center of Excellence for Drug Preclinical Studies (CE-DPS), Pharmaceutical and Fermentation Industry Development Center, City of Scientific Research & Technological Applications, New Borg El Arab, Alexandria, Egypt.

[3] Bio-screening and Preclinical Trial Lab, Biochemistry Department, Faculty of Science, Alexandria University, Alexandria, Egypt.

[4] Biochemistry Department, Faculty of Science, Alexandria University, Alexandria, Egypt.

*Author of correspondence: Dr. Alaa A. El-Banna, Alkhartoom square, Department of Pharmacognosy, Faculty of Pharmacy, Alexandria University, Alexandria, 21521, Egypt.

E-mail: alaaelbanna32@yahoo.com.

E-mail: alaa.elbanna@alexu.edu.eg

**Supplementary Table S1. XP G scores of *L. camara* top hit compounds in the compound–target network against the most enriched inflammation-associated target proteins.**

| | **Protein kinase C alpha type (4RA4)** | **Transcription factor p65 (3QXY)** | **Interleukin-2 (1M49)** | **Mitogen-activated protein kinase 14 (6HWU)** | **Proto-oncogene c-Fos (1FOS)** |
|---|---|---|---|---|---|
| Ferulic acid | -5.383 | -2.237 | -5.602 | -8.770 | -4.751 |
| Catechin gallate | -9.898 | -4.660 | -6.664 | -9.901 | -8.454 |
| Myricetin | -8.648 | -3.933 | -6.014 | -11.928 | -10.030 |
| Isoferulic acid | -7.710 | -3.489 | -5.187 | -8.206 | -4.601 |

**Supplementary Table S2. Enrichment calculations for the investigated most enriched target proteins**

| **Enzyme**<br>**PDB code** | **Protein kinase C alpha type (4RA4)** | **Transcription factor p65 (3QXY)** | **Interleukin-2 (1M49)** | **Mitogen-activated protein kinase 14 (6HWU)** | **Proto-oncogene c-Fos (1FOS)** |
|---|---|---|---|---|---|
| **AUC-ROC** | 0.992 | 0.992 | 0.995 | 0.992 | 0.989 |
| **EF(2%)** | 48 | 48 | 50 | 48 | 43 |
| **EF(5%)** | 19 | 19 | 20 | 19 | 19 |
| **EF(10%)** | 9.4 | 9.4 | 10 | 9.4 | 9.6 |
| BED-ROC | | | | | |
| ***α* = 160.9** | *0.987* | *0.987* | *1* | *0.987* | 0.996 |
| ***α* =20** | *0.950* | *0.950* | *1* | *0.950* | *0.967* |
| ***α* =8** | 0.945 | *0.945* | *1* | *0.945* | *0.962* |
| **No. of actives** | 16 | 17 | 13 | 16 | 23 |
| **Ranked actives** | 15 | 16 | 13 | 15 | 22 |
| **RMSD** | **1.172** | **----------** | **0.386** | **0.558** | **----------** |

**Supplementary Table S3. ADME characteristics of *L. camara* top hit compounds**

| Compound | mol_MW | donorHB | accptHB | QPlogPo/w | Percent Human Oral Absorption |
|---|---|---|---|---|---|
| Myricetin | 318.239 | 5 | 6 | -0.299 | 37.45 |
| Catechin gallate | 442.378 | 7 | 8 | 0.476 | 36.903 |
| Ferulic acid | 194.187 | 2 | 3.5 | 1.375 | 67.296 |
| Isoferulic acid | 194.187 | 2 | 3.5 | 1.373 | 67.251 |

**Supplementary Table S4. Brief literature survey on the top scoring *L. camara* constituents as anti-inflammatory candidates.**

| Compound | Inflammation-associated diseases | Model | PMID number | Mechanism |
|---|---|---|---|---|
| 1. Ferulic acid | Pre-eclampsia | NG-nitro-l-arginine methyl ester (L-NAME)-induced rats | 30183401 | - Decreased expression of circulating TNF-*α*、IL-6, IL-1*β* and PlGF.<br>- Reduced placental TNF-α and NF-κB p65.<br>- Rescued decreasing expression of IL-4 and IL-10 in the circulation and placenta of rats.<br>- Ameliorated placental apoptosis by increasing Bcl-2 and decreasing Bax expression in placenta. |
| | Diabetic neuropathy | Streptozotocin-administered rats | 30804780 | - Modulated AGEs, MAPKs (p38, JNK, and ERK 1/2), NF-κB mediated inflammatory pathways, mitochondria-dependent and -independent apoptosis as well as autophagy induction. |
| | Hepatotoxicity | Methotrexate (MTX) - administered rats | 31889292 | - Reduced serum TNF-*α* and IL-1*β*, and hepatic NF-κB p65, Bax, and caspase-3, whereas increased Bcl-2, Nrf2, NQO1, HO-1, and PPAR*γ* |
| | Endometritis | LPS stimulated bovine endometrial epithelial cells (BEECs) | 31499425 | - Reduced mRNA expression of LPS-induced proinflammatory cytokines (IL1*β*, IL6, TNFA, and IL8) Inhibited the degradation of IκB and phosphorylation of NF-κB p65.<br>- Suppressed the phosphorylation of MAPKs, including p38 and JNK. |

| | | | | |
|---|---|---|---|---|
| | Testicular inflammation | cadmium chloride ($CdCl_2$ )-administered male rats. | 33047361 | - alleviated apoptotic and inflammatory injuries in testicular tissue via Nrf2 activation. |
| | Acute respiratory distress syndrome (ARDS) | LPS administered rats | 29350567 | - It up-regulated the secretion of interleukin IL-1$\beta$, IL-6, tumor necrosis factor TNF-$\alpha$, and IL-10 in BALF cells |
| 2.Catechin gallate | Pancreatic cancer | human pancreatic ductal adenocarcinoma (PDAC) cells: PancTu-I, Panc1, Panc89 and BxPC3 | 21241417 | - Inhibited TNFα-induced activation of NF-κB and consequently secretion of pro-inflammatory and invasion promoting proteins like IL-8 and uPA. |
| | UV radiation - induced erythyma | healthy humans (phototype I/II) | 23351338 | - Reduced levels of cyclo-oxygenase- and lipoxygenase-produced mediators of UVR inflammation, PGE2 and 12 -hydroxy-eicosatetraenoic acid (12-HETE), respectively. |
| 3.Myricetin | Non-alcoholic steohepatitis (NASH) | - C57BL/6J mice<br>- NASH mice<br>-RAW264.7 macrophages | 32195263 | - Modulated the polarization of macrophages via inhibiting the TREM-1-TLR2/4-MyD88 signaling molecules in macrophages |
| | Acute lung injury | - LPS -stimulated RAW 264.7 cells<br>- LPS-induced lung injury model. | 30095283 | - Suppressed the NF-κB p65 and AKT activation in NF-κB pathway and JNK, p-ERK and p38 in MAPK signaling pathway. |
| | Pathological cardiac hypertrophy | - Wild type (WT) and cardiac Nrf2 knockdown (Nrf2-KD) mice | 31885808 | - Increased Nrf2 activity, decreased NF-$\kappa$B activity, and inhibited TAK1/p38/JNK1/2 MAPK signaling |
| | colorectal tumorigenesis | AOM/DSS administered rats | 29136951 | - Abolished the levels of inflammatory factors TNF-$\alpha$, IL-1$\beta$, IL-6, NF-κB, p-NF-κB, cyclooxygenase-2 (COX-2), PCNA and Cyclin D1 in the colonic tissues |
| | Mastitis | Mice mammary epithelial cells (mMECs)<br>LPS administered lactating female rats | 30746687 | - Decreased activity of myeloperoxidase (MPO) and the production of TNF-$\alpha$, IL-6, and IL-1$\beta$ triggered by LPS<br>- Inhibiting LPS-induced phosphorylation of AKT, IKK-$\alpha$, IκB-α, and P65<br>- inhibiting the AKT/IKK/NF-κB signaling pathway. |

| | | | | |
|---|---|---|---|---|
| | Nephrotoxicity | Cisplatin administered rats | 28064632 | - Decreased caspase-3, TNF-$\alpha$, IL-6, COXI and COXII, MDA levels |
| | DM-associated kidney injury | wild-type (WT) and Nrf2 knockdown (Nrf2-KD) mice | 31244660 | - Prevented DM-associated decreased expression of Nrf2 and inhibited IκB/NF-κB (P65) signaling pathway. |
| 4.Isoferulic acid | Rheumatism | LPS-stimulated human blood | 19935904 | - Inhibited levels of IL-6, TNF-$\alpha$, and IFN-$\gamma$. |
| | Neuritis | LPS-stimulated BV2 microglial cells | 24291391 | - Suppressed NO and $PGE_2$ production through the induction of nuclear factor erythroid 2-related factor 2 (Nrf2)-dependent heme oxygenase-1 (HO-1). |
| | Respiratory Syncytial Virus Infection | murine macrophage cell line (RAW264.7) | 10704056 | - Inhibited the production of macrophage inflammatory protein-2 (MIR-2) |

**Supplementary Table S5. Detailed interactions of the highest scoring compounds with their respective target proteins**

| Compound | Protein target | Interactions | |
|---|---|---|---|
| | | Type | Amino acid residues (distances, A°) |
| **Catechin gallate** | **Protein kinase C alpha type (4RA4)** | Hydrogen bonds | GLU418(1.34,1.37), ASP424 (1.04,1.08) |
| | | Hydrophobic | PHE350, ALA480, MET417, TYR419, VAL420, ALA366, MET470, LEU345, VAL353 |
| | **Transcription factor p65 (3QXY)** | Hydrogen bonds | LEU362(1.23,1.25), ARG208(1.34), GLU197(1.57, 1.64) |
| | | Hydrophobic | LEU362, LEU205, LEU193, TYR188, LEU210 |
| | **Interleukin-2 (1M49)** | Hydrogen bonds | ARG38(0.94,0.96,1.64,1.65), LYS43(1.01), GLU68(1.47) |
| | | Hydrophobic | MET39, PHE42, PRO65, PHE44 |
| **Myricetin** | **Mitogen-activated protein kinase 14 (6HWU)** | Hydrogen bonds | ALA51(1.34), MET109(1.09,1.53), VAL30(1.09) |
| | | Hydrophobic | VAL30, VAL38, VAL52, VAL105, ALA51, LEU104, LEU75, PHE169, LEU108, MET109, LEU171 |
| | **Proto-oncogene c-Fos (1FOS)** | Hydrogen bonds | SER154(1.21), SER278(1.01,1.34), ARG158 (1.32), DG30(1.56), DG8(1.24), DA9(1.29) |
| | | Hydrophobic | ALA151 |

**Supplementary Table S6: Structural features of active compounds used in enrichment studies for each of the investigated target proteins**

| Protein kinase C alpha type (4RA4) | | | | | | | | |
|---|---|---|---|---|---|---|---|---|
| # | name | Structure | # | name | Structure | # | name | Structure |
| 1. | Calphostin [1] | 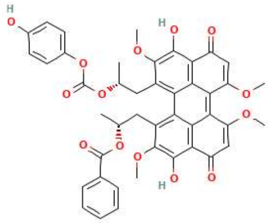 | 2. | Enzastaurin [1] | 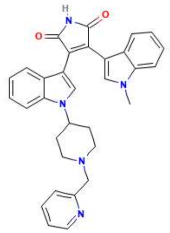 | 3. | Riluzole [1] | 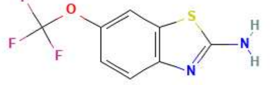 |
| 4. | Staurosporine [1] | 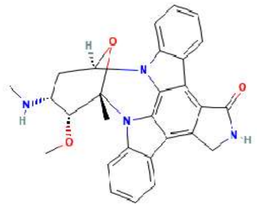 | 5. | Tamoxifen [2] | 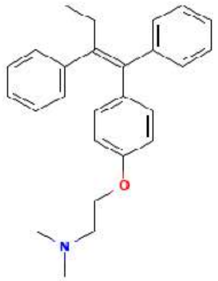 | 6. | Ruboxistaurin [3] | 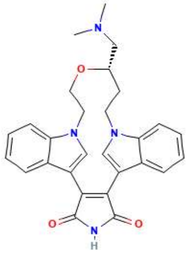 |
| 7. | 7-hydroxystaurosp-orine [1] | 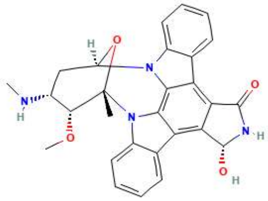 | 8. | N-benzyladriamycin-14-valerate [1] | 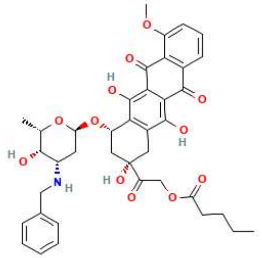 | 9. | Safingol [1] | 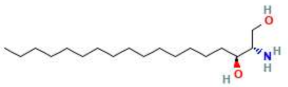 |

| | | | | | | | | |
|---|---|---|---|---|---|---|---|---|
| 10. | Auranofin [1] | 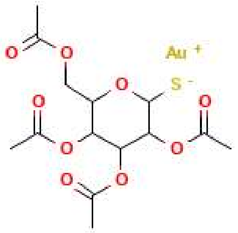 | 11. | Resveratrol [1] | 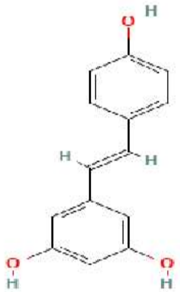 | 12. | Sodium aurothiomalate [1] | 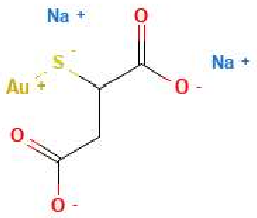 |
| 13. | NPC-15437 [1] | 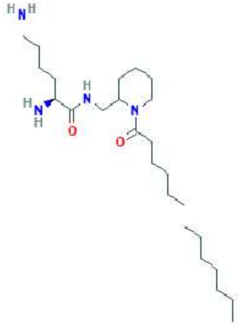 | 14. | Sotrastaurin [1] | 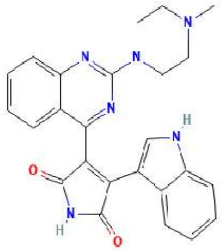 | 15. | Balanol [1] | 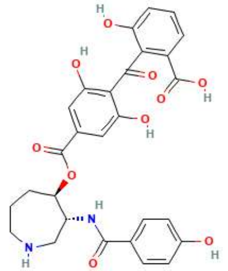 |
| 16. | Chelerythrine [1] | 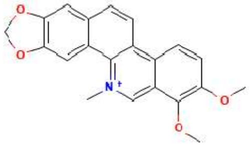 | | | | | | |
| **Transcription factor p65 (3QXY)** | | | | | | | | |
| 1. | Glusodichoto-mine AK [4] | 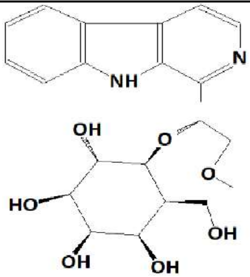 | 2. | 3-hydroxy-β-carboline [4] | 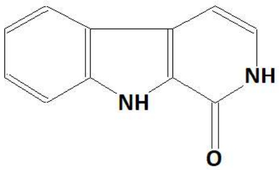 | 3. | Arenarine D [4] | 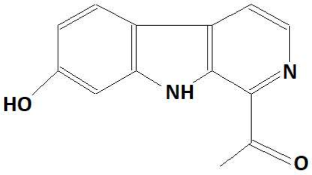 |

| | | | | | | | | |
|---|---|---|---|---|---|---|---|---|
| 4. | β-carboline-1-carboxylic acid [4] | 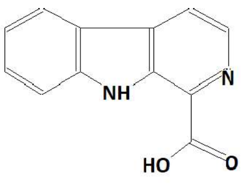 | 5. | 1,2,3,4 dihydro-1,3,4-trioxo-β-carboline [4] | 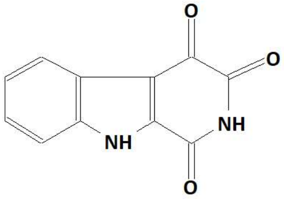 | 6. | Arenarine B [4] | 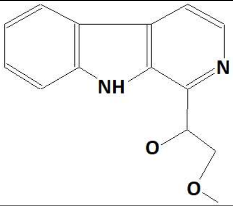 |
| 7. | Trichostatin A [5] | 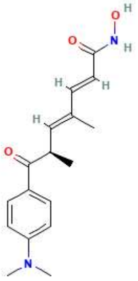 | 8. | Barbaloin [6] | 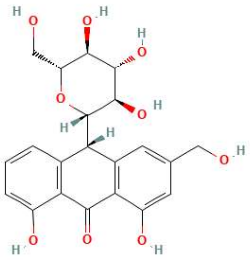 | 9. | Ganoderic acid [7] | 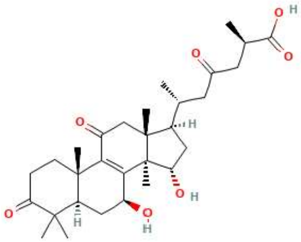 |
| 10. | Curcumin [8] | 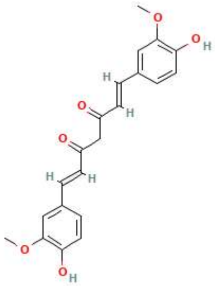 | 11. | Caffeic acid phenethyl amide (CAPA) [9] | 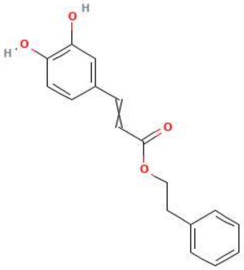 | 12. | Convallatoxin [10] | 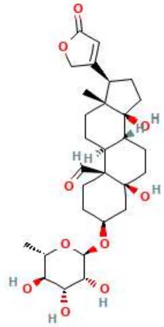 |
| 13. | Lisinopril [11] | 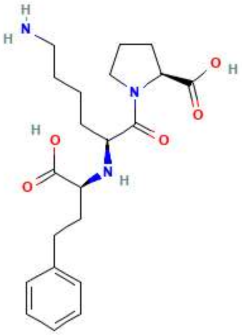 | 14. | Carnosol [12] | 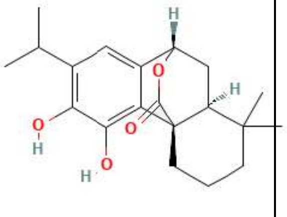 | 15. | Dihydroartemisinin [13] | 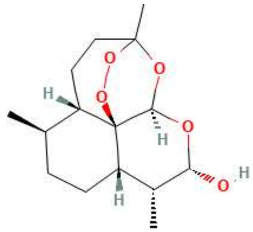 |

| | | | | | | | | |
|---|---|---|---|---|---|---|---|---|
| 16. | Silymarin [11] | 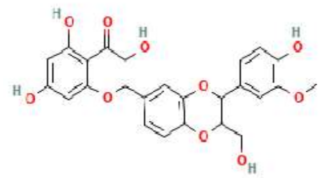 | 17. | DOPA [14] | 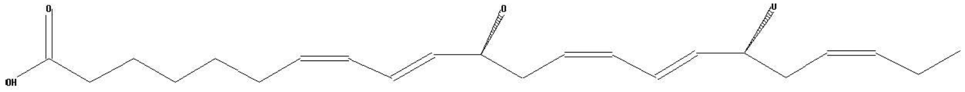 | | | |
| **Interleukin-2 (1M49)** | | | | | | | | |
| 1. | 1[15] | 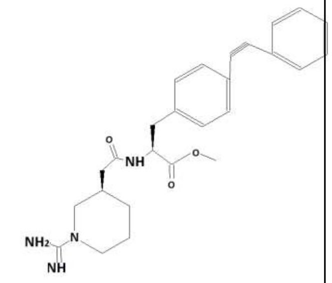 | 2. | Nectandrin A[16] | 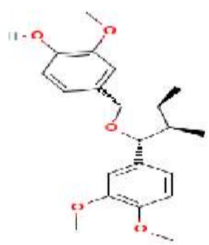 | 3. | Galgravin[16] | 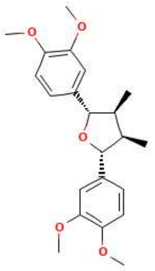 |
| 4. | 2 [15] | 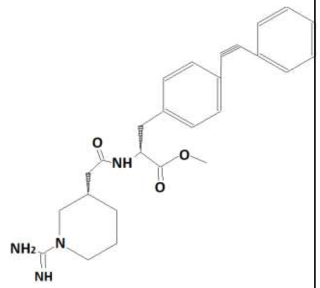 | 5. | $A_{17}B_{284}$ [17] | 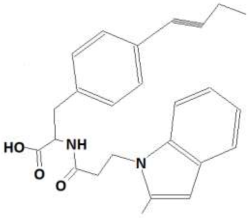 | 6. | Pentoxifylline [18] | 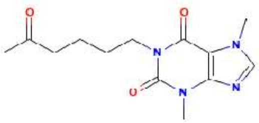 |
| 7. | 1[19] | 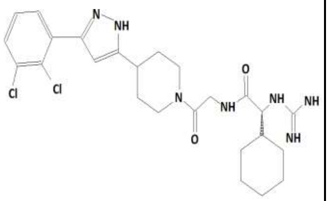 | 8. | Dexamethasone [20] | 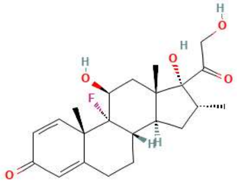 | 9. | Trichostatin A[21] | 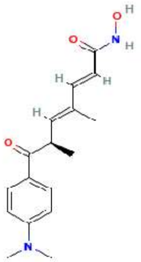 |

| | | | | | | | | |
|---|---|---|---|---|---|---|---|---|
| 10. | 2 [19] | 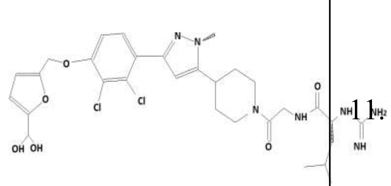 | 11. | futokadsurin[16] | 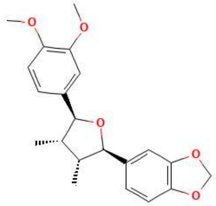 | 12. | Tyrphostin [22] | 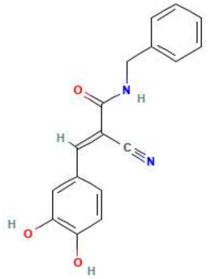 |
| 13. | Veraguensin[16] | 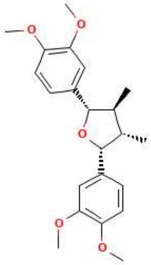 | | | | | | |
| **Mitogen-activated protein kinase 14 (6HWU)** | | | | | | | | |
| 1. | Pamapimod [23] | 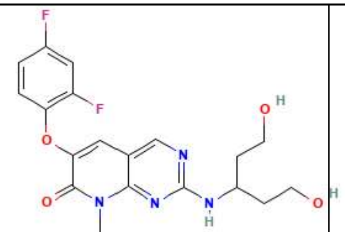 | 2. | FR 167653 [24] | 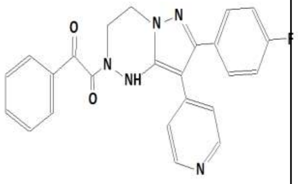 | 3. | Montelukast [25] | 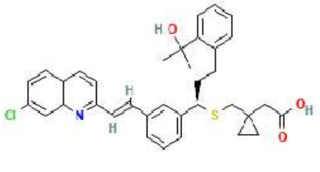 |
| 4. | Genistein [26] | 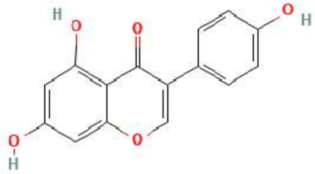 | 5. | BIRB 795 [27] | 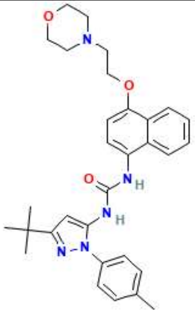 | 6. | SB239063 [28] | 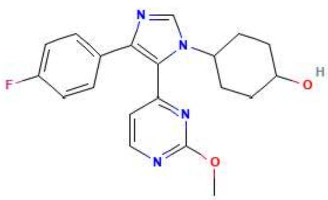 |

| | | | | | | | | |
|---|---|---|---|---|---|---|---|---|
| 7. | SB202190 [29] | 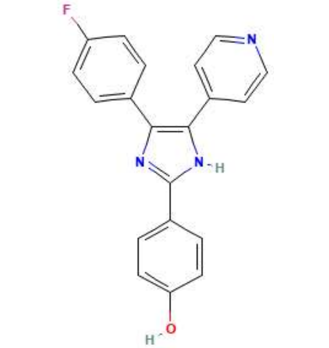 | 8. | SB203580 [30] | 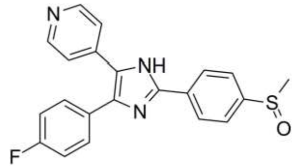 | 9. | Losmapimod [31] | 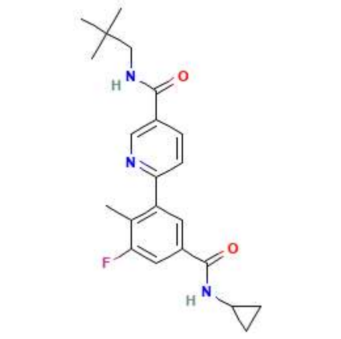 |
| 10. | Skepinone-L [32] | 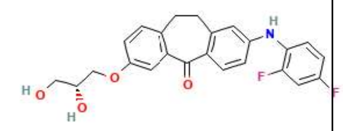 | 11. | Na salicylate [33] | 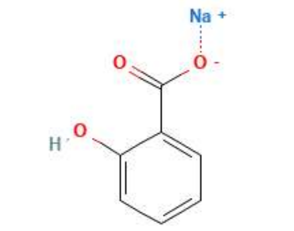 | 12. | SCIO-469 [34] | 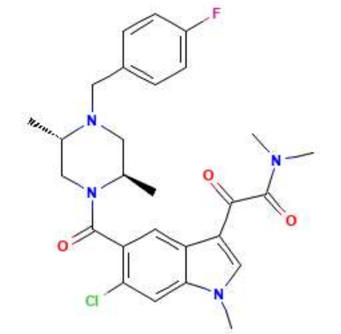 |
| 13. | Ralimetinib [35] | 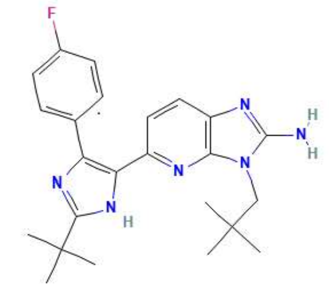 | 14. | RWJ 67657 [36] | 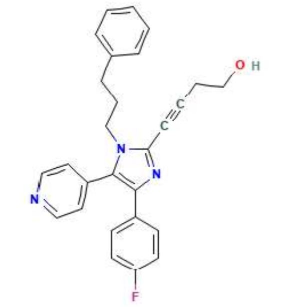 | 15. | VX-702 [37] | 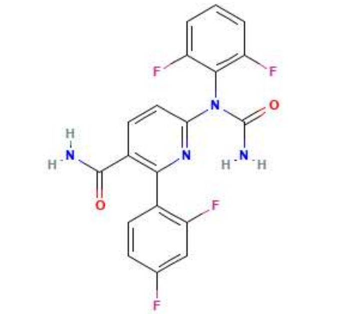 |

| | | | | | | | | |
|---|---|---|---|---|---|---|---|---|
| 16. | BMS-582949 [38] | 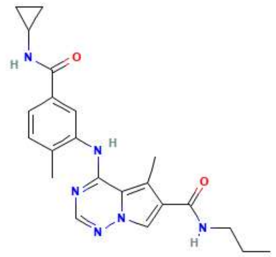 | | | | | | |
| **Proto-oncogene c-Fos (1FOS)** | | | | | | | | |
| 1. | momordin[39] | 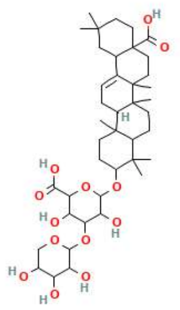 | 2. | Acteoside[40] | 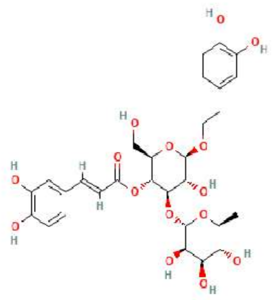 | 3. | Nagilactone [39] | 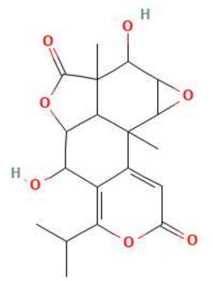 |
| 4. | Curcumin [39] | 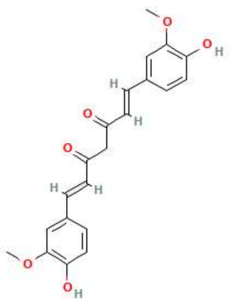 | 5. | Kaempferol[41] | 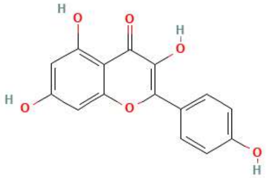 | 6. | Citrifolinin A[39] | 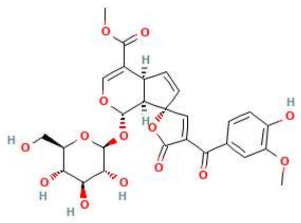 |

| | | | | | | | | |
|---|---|---|---|---|---|---|---|---|
| 7. | Grassypeptolide F [39] | 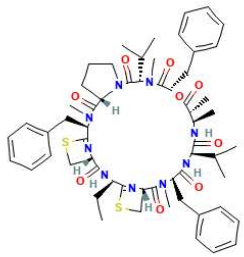 | 8. | Grassypeptolide G [39] | 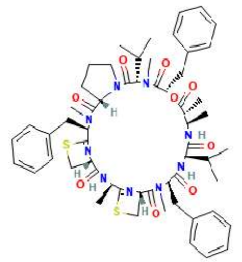 | 9. | Dihydroguaiaretic acid [39] | 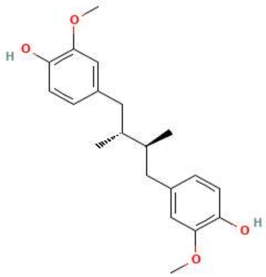 |
| 10. | Citrifolinoside [39] | 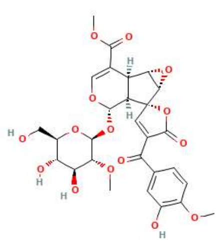 | 11. | Sch 56396 [42] | 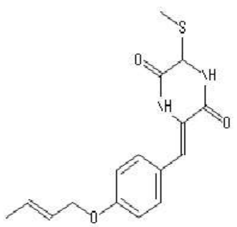 | 12. | SP100030 [39] | 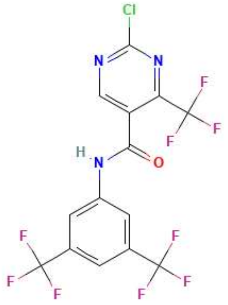 |
| 13. | Inumakilactone [39] | 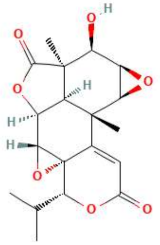 | 14. | SPC-839 [39] | 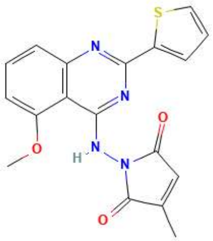 | 15. | Senecionylchaparrin [39] | 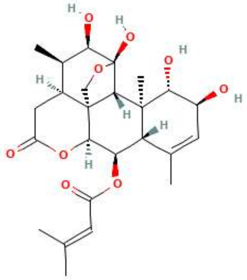 |

| | | | | | | | | |
|---|---|---|---|---|---|---|---|---|
| 16. | Glaucarubinone [39] | 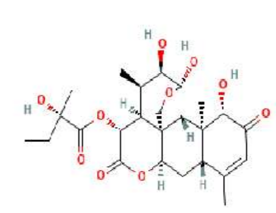 | 17. | Isosteviole [39] | 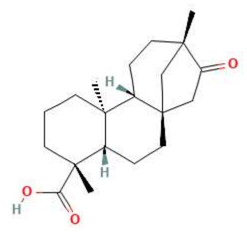 | 18. | Isosteviole lactone [39] | 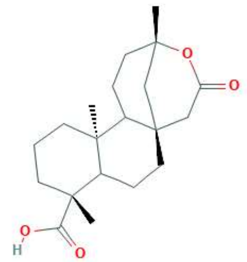 |
| 19. | Ailanthinone [39] | 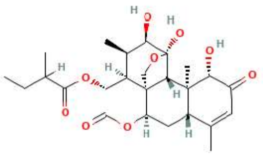 | 20. | T-5224 [43] | 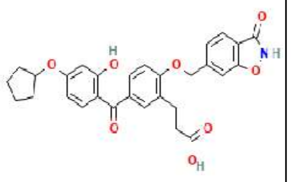 | 21. | K1115 A [39] | 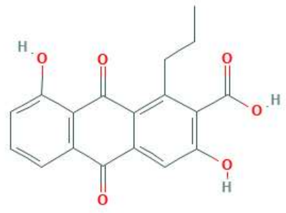 |
| 22. | 6-gingerol [39] | 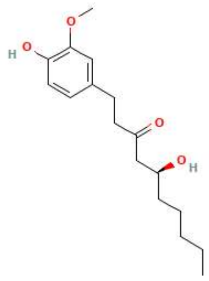 | 23. | 6-shogaol [39] | 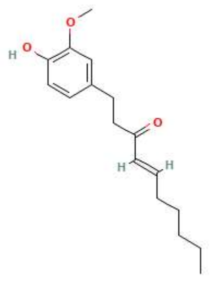 | | | |

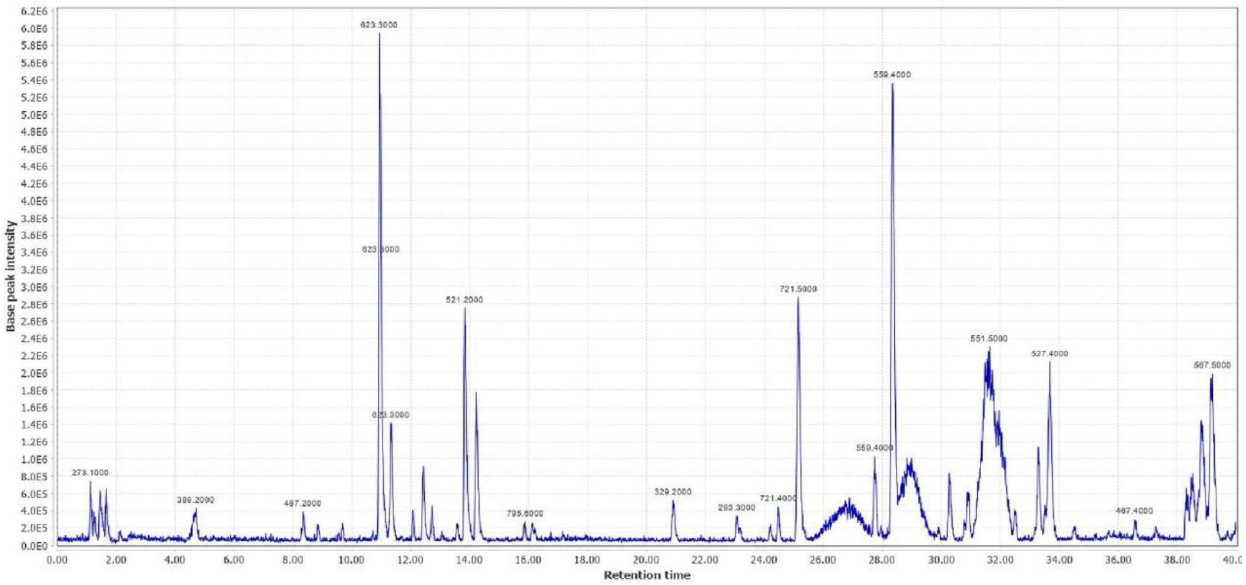


**Supplementary Figure S1.** Base peak chromatogram of the extract of *L. camara* leaves.

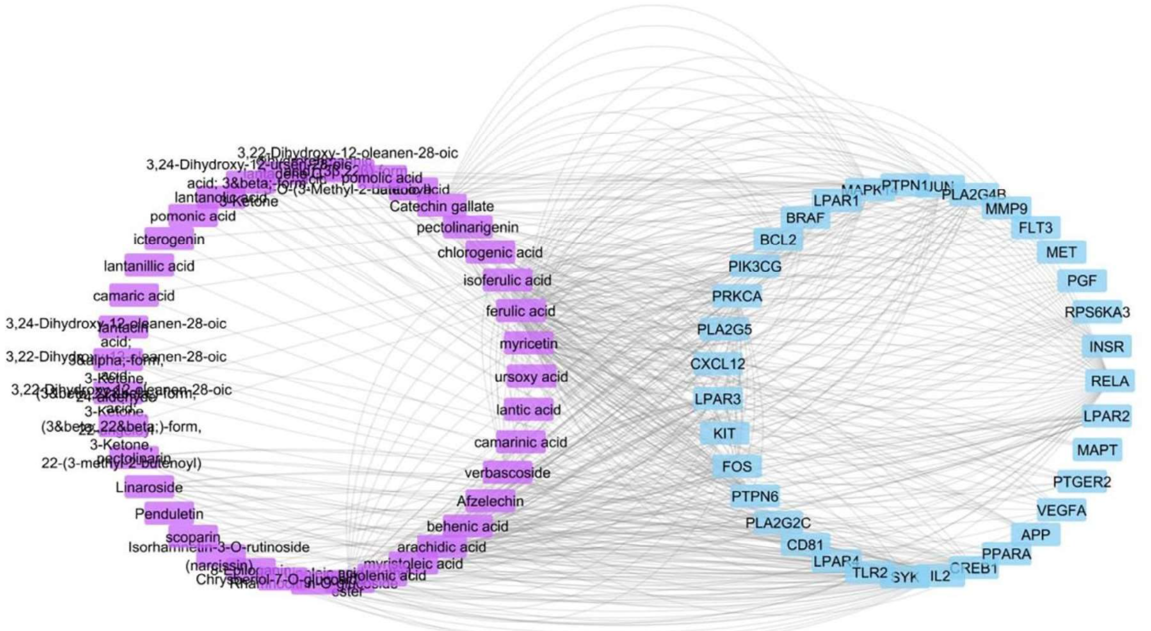


**Supplementary Figure S2.** Network of compound–target gene interactions for *L. camara* constituents by linking 39 compounds (presented in violet color) and 35 target proteins (presented in blue color).

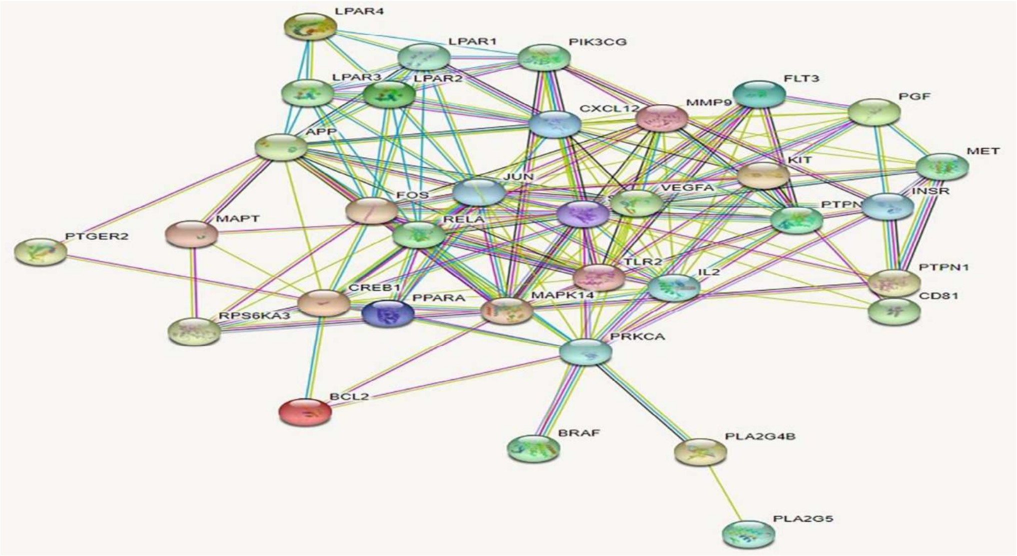


**Supplementary Figure S3.** Protein–protein interaction (PPI) network of identified inflammation-related targets.

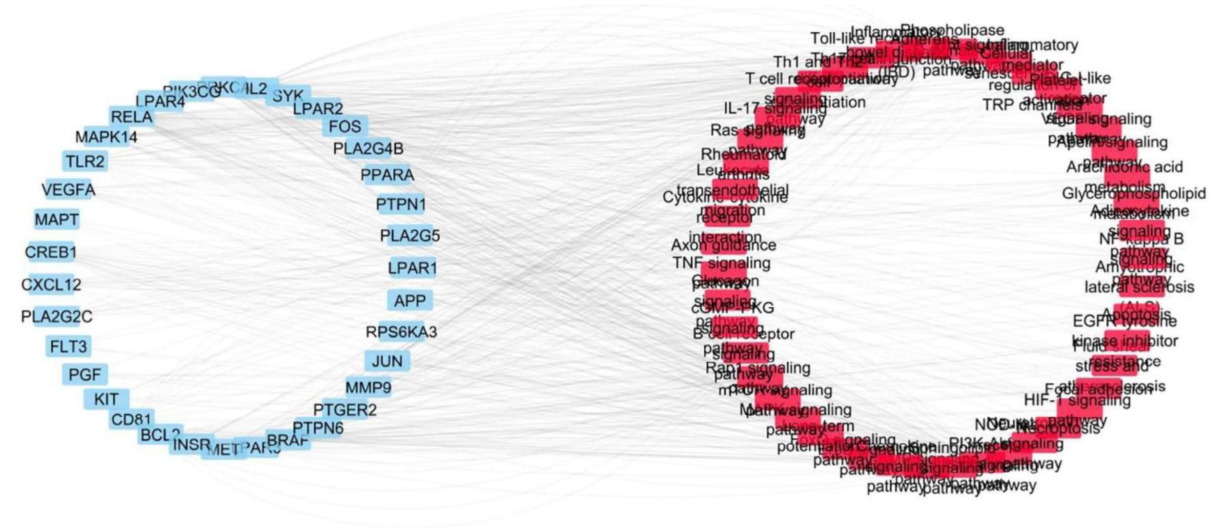


**Supplementary Figure S4.** Gene–pathway network (genes are presented in blue color, pathways are presented in red color).

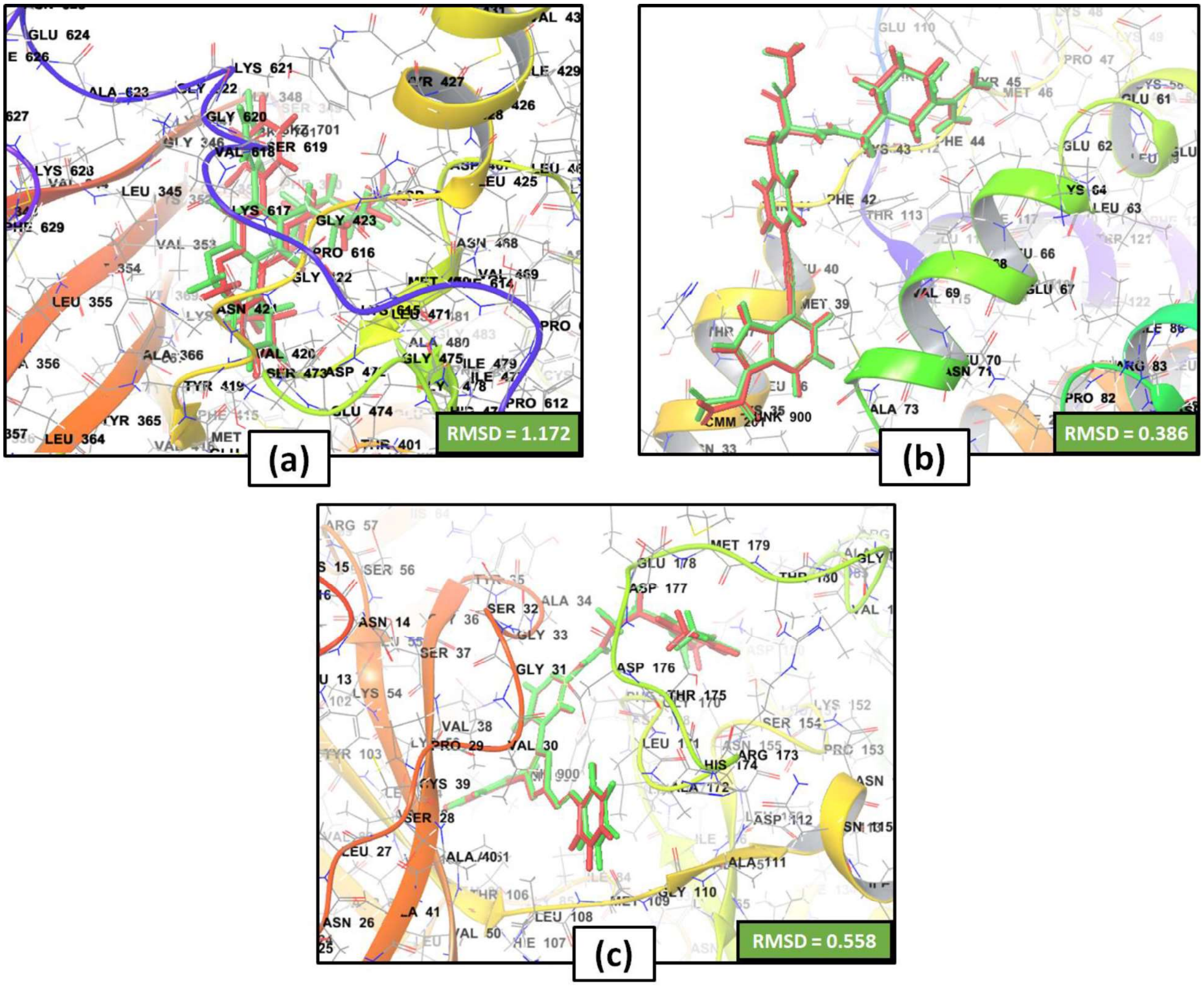


**Supplementary Figure S5**. Superimposition of co-crystallized ligand (red) and re-docked ligand (green) at the binding site of the proteins (a) Protein kinase C alpha type (4RA4), (b) interleukin-2 (1M49) and (c) Mitogen-activated protein kinase 14 (6HWU).

# Methods:

### Analysis of *L. camara* extract using UPLC-MS/MS technique:

### ESI-MS conditions and metabolites annotation:

Electrospray ionization (ESI) source in conjunction with triple quadrupole (TQD) mass spectrometer were utilized to analyze the samples in a negative ionization mode. ESI operating conditions briefly were: capillary voltage of 3 kV, cone voltage; 35 V, the ion source temperature was 150°C, the nebulizer (nitrogen gas) pressure was 35 psi, drying and sheath gas ($N_2$) temperatures were 440°C and 350°C, respectively. The drying and sheath gas flows were applied at 900 L/h and 50 L/h, respectively. The analytical run time was extended to 30 min. MS spectra were achieved by full range acquisition covering 100-1000 m/z. For automatic MS/MS fragmentation analyses of the precursor ions which were mass-selected by the first quadrupole (Q1), the collision-induced dissociation (CID) energy was ramped from 30 to 70 eV using nitrogen gas as a collision gas in the second quadrupole collisional cell (Q2). Finally, the daughter ions yielded from CID are consequently related to the molecular structure of the precursor ions and can be monitored by a third quadrupole mass analyzer (Q3). Assignment of the metabolites was accomplished by comparison of their retention times to external standards. Furthermore, quasi-molecular ions in addition to the characteristic MS/MS fragmentation pattern were used for metabolite annotation in comparison to our in-house database, reference literature and phytochemical dictionary of natural products database (CRC) in order to get metabolite annotation with a high level of confidence [44].

### *In vitro* cytotoxicity and anti-inflammatory activity testing

It was carried out according to the method described by Darwish et al.[45] as following:

### Isolation and cultivation of human white blood cells

A blood specimen was provided from Alexandria Regional Blood Transfusion Center (63 Ahmed Soliman El-Shaikh Street, Kom Ad Dakah Sharq, Al Attarin, Alexandria Governorate, Egypt). This blood specimen was placed in a sterile heparin tube, from which 1 mL was drawn into 15 mL centrifuge tube which was then filled with 10%v/v fresh cold lysing solution prepared from stock solution containing NH4Cl 8.02g, NaHCO3 0.84g and EDTA 0.37g. The centrifuge tube was then inverted at room temperature for about 10 min till the liquid turned into clear red. After that, the

blood specimen was centrifuged at 2000 rpm and 4° C for 10 min and the supernatant was decanted. The pellets (WBCs) were suspended in 10 mL cold PBS (phosphate-buffered saline containing 137 mM NaCl, 2.7 mM KCL, 10 mM $Na_2HPO_4$ and 10 mM $KH_2PO_4$, pH 7.4) and centrifuged. Then they were resuspended in RPMI-1640 medium, containing 10% fetal bovine and 2% L-glutamine. The evaluation of WBCs viability and counting was carried out using dye exclusion method[46]. Fifty µL of cell suspension was blended with equal volume of 0.5% trypan blue staining solution then loaded onto hemocytometer. Eventually, counting of viable "unstained" and nonviable "stained" cells in each of the four corner quadrants (A, B, C, D) was carried out.

**Calculation**

N / mL = mean of WBCs counting x$10^4$ × D

N: Number of viable or nonviable cells

D: Sample dilution (1:1 with the trypan blue).

% Cell viability = (Number of viable cells / Total number of cells) × 100

% Cell viability must be at least 90% in order to perform the assays. WBCs were cultured in RPMI media and incubated in $CO_2$ incubator at 37°C, 5% $CO_2$, and 90% relative humidity for six days. Then they were seeded in 96 well cell culture plate (100,000 cells/ well).

**Assessment of cytotoxicity of the crude extracts compared to piroxicam (MTT assay)**

Treatment of 200 µL cultured medium containing 100,000 WBCs / well with different concentrations (0, 3.125, 6.25, 12.5, 25 and 50 µg/mL) of the crude extracts was carried out in RPMI medium without fetal bovine serum, using the known NSAID drug 'Piroxicam' as a positive control. Plates were then incubated in $CO_2$ incubator for 72 h in the same conditions, then 20 µL of MTT solution was added to each well and re-incubated to allow MTT reaction to be accomplished. Afterwards, the plates were centrifuged at 1650 rpm for 10 min and the medium was discarded. The MTT byproducts (formazan crystals) were suspended in 100 µL DMSO and measurement of absorbance was performed at a wavelength of 570 nm using optima spectrophotometer, in order to detect the safe dose which causes 100% cell viability.

The % viability was calculated as follow: ($A_T$-$A_b$ /$A_C$-$A_b$) x 100

$A_T$ = mean absorbances of cells treated with a certain concentration of the plant extract.

$A_C$ = mean absorbances of control untreated cells with culture medium only

$A_{b=}$ mean absorbances of cells treated with vehicle of plant extract (RPMI without fetal bovine serum)

The cytotoxicity assay of the compound was expressed as $CC_{50}$, which is the drug concentration required for reducing the cell viability by 50%, and it was calculated by the Graphpad Instat software (https://www.graphpad.com/scientific-software/instat/) by interpolation from the plot of % cell viability vs serial dilutions of the plant extract.

**Detection of the effective anti-inflammatory concentrations (EAICs) of the tested extract in lipopolysaccharides (LPS)-stimulated human WBC's culture**

In a 96 well plate, dispensing of 50 µL of the culture medium containing 100,000 of human WBCs per well was carried out. Induction of inflammation was accomplished by adding 50 µL of LPS to the plated cell, then they were incubated in $CO_2$ incubator. After 24 h, centrifugation of the plate at 1650 rpm for 5 min was performed, then the supernatants were discarded. Afterwards, addition of 200 µL of serial concentrations (0, 3.125, 6.25, 12.5, 25 and 50 µg/mL in culture media) of the crude extracts or the standard anti-inflammatory drug piroxicam was carried out. The blank was wells containing untreated cells in culture medium only. The plates were then incubated for another 72 h in $CO_2$ incubator. Thereafter, measurement of cells proliferation was carried out using MTT test and it was expressed as stimulation index (SI).

**Stimulation index** = (mean absorbance of LPS-stimulated cells (negative control) or LPS-stimulated cells treated with different concentrations of plant extract / absorbance of control untreated cells (blank)).

Graphpad Instat software was used to calculate the effective anti-inflammatory concentration (EAIC) of the extract that was able to resume the abnormal proliferation of LPS-stimulated cells to normal proliferation of control untreated cells (SI = 1)

**Extraction of RNA of untreated and treated LPS-stimulated human white blood cells**

Cell pellets were suspended in 50 µL of solution R1 (qiagen RNA extraction kit). They were mixed for 30 s, then incubated for 1 min at room temperature. Afterwards, 300 µL of solution R2 (qiagen RNA extraction kit) were added and blended for 30 s. Centrifugation was accomplished for 3-5

min at 4ºC. Next, the supernatant was transferred into a spin column and centrifuged at 14000 rpm for 30 s at 4ºC. Then, addition of 300 µL of working wash buffer was done after removing the flow-through and centrifugation was repeated for 30 s. This step was repeated twice. Subsequently, the spin column was centrifuged at 10,000 rpm for 1 min then delivered to a sterile 1.5 mL micro centrifuge tube. Then, 30 µL of elution buffer were added to the membrane center and incubated for 1 min at room temperature, then centrifuged at 14000 rpm for 30 s at 4ºC. Eventually, the optical density (OD) of the extracted RNA was determined via absorbance and purity measurement at A260 and A260/A280 nm, respectively. Then it was kept at -80°C until real time polymerase chain reaction (PCR) was performed.

**cDNA synthesis from RNA extracted from untreated and treated LPS-stimulated human white blood cells**

In PCR tubes, 2 µg of total RNA or nuclease-free water and 1 µL of oligo dT primer were added to nuclease-free water in a total volume of 12 µL, and they were gently mixed. Centrifugation was performed before incubation for 5 min at 65ºC in PCR machine, then the mixture was set immediately on ice. Four microliters of 5X reaction buffer, 1 µL of RNase inhibitor, 2 µL of dNTPs mix and 1 µL of reverse transcriptase or 1 µL of nuclease-free water instead of reverse transcriptase for reverse transcriptase negative control were gently mixed with previous mixture. Afterwards, the PCR tubes were spined down and incubated for 60 min at 42ºC, then they were heat inactivated at 70ºC for 5 min in PCR machine

**Determination of IL-1*β*, IL 6, TNF and INF-*γ* expression level by real time polymerase chain reaction (PCR)**

In PCR tubes, admixture of 13 µL of 2 X SYBR green master mix with 5 µL of cDNA, 0.5 µL of 10 pmoles/mL forward primer and 0.5 µL of 10 pmoles/mL reverse primer for each primer was carried out. The same as in the reference tube, addition of 0.5 µL of 10 pmoles/mL forward primer of β-actin and 0.5 µL of 10 pmoles/mL for reverse primer of β- actin was done. Another tube was utilized as a non-template control (NTC) to assess reagent contamination or primer dimers, by inserting 1 µL of nuclease-free water as a substitute of template used. Afterwards, the tubes were subjected to gentle mixing with 6.5 µL nuclease free water without bubbles formation and subsequently subjected to spinning for few seconds. Samples were set in the cycler and the program was initiated as following; initial denaturation (1 cycle of 95ºC for 10 min), then

denaturation (40 cycles of 95ºC for 15 sec), annealing (at 60ºC for 30s) and extension (at 72ºC for 30s). Fold change in gene expression was used to assess the influence of LPS and extracts on the expression of genes.

**Calculation**

**Expressions fold levels of gene are computed by**

$\Delta Ct_{\text{normal}} = Ct_{\text{normal untreated cells}} - Ct_{\text{reference}}$

$\Delta Ct_{\text{tested plant extract}} = Ct_{\text{tested plant extract-treated cells}} - Ct_{\text{reference}}$

$\Delta Ct_{\text{induced}} =- Ct_{\text{LPS-exposed cells}} - Ct_{\text{reference}}$

**In case of genes:**

$\Delta\Delta CT_{\text{tested plant extract}} = \Delta Ct_{\text{tested plant extract}} - \Delta Ct_{\text{normal}}$

$\Delta\Delta CT_{\text{induced}} = \Delta Ct_{\text{induced}} - \Delta Ct_{\text{normal}}$

**In case of GAPDH:**

$\Delta\Delta CT_{\text{tested plant extract}} = \Delta Ct_{\text{normal}} - \Delta Ct_{\text{tested plant extract}}$

$\Delta\Delta CT_{\text{induced}} = \Delta Ct_{\text{normal}} - \Delta Ct_{\text{induced}}$

**Fold change in gene expression = log ($2^{-\Delta\Delta CT}$)**

**Where:**

**$Ct_{\text{tested plant extract}}$**: threshold cycle value of genes of extracted mRNA of plant extract treated-LPS-stimulated WBCs which is defined as the cycle number at which the fluorescence generated within a reaction crosses the fluorescence threshold.

**$Ct_{\text{reference}}$**: threshold cycle value of GAPDH which is used for normalization.

**$Ct_{\text{normal}}$**: threshold cycle value of genes of extracted mRNA of untreated control WBCs

**$Ct_{\text{induced}}$**: threshold cycle value of gene of extracted mRNA of LPS-stimulated WBCs

Primers

| | |
|---|---|
| TNF-$\alpha$ | F-CTCTTCTGCCTGCTGCACTTTG |
| | R- ATGGGCTACAGGCTTGTCACTC |
| IL-6 | F, 5′-TGAACTCCTTCTCCACAAGCG-3′ |
| | R, 5′-TCTGAAGAGGTGAGTGGCTGTC-3′ |
| IL-1$\beta$ | F, CCACAGACCTTCCAGGAGAATG |
| | R, GTGCAGTTCAGTGATCGTACAGG |

| | |
|---|---|
| INF-$\gamma$ | F, GAGTGTGGAGACCATCAAGGAAG |
| | R, TGCTTTGCGTTGGACATTCAAGTC |
| GAPDH | F, GGATTTGGTCGTATTGGG |
| | R, GGAAGATGGTGATGGGATT |

## References:


1. Kawano, T., Inokuchi, J., Eto, M., Murata, M. & Kang, J.-H. Activators and Inhibitors of Protein Kinase C (PKC): Their Applications in Clinical Trials. *Pharmaceutics* **13**, 1748 (2021).
2. Zarate, C. A. & Manji, H. K. Protein kinase C inhibitors: rationale for use and potential in the treatment of bipolar disorder. *CNS Drugs* **23**, 569–582 (2009).
3. Dowey, R. *et al.* Enhanced neutrophil extracellular trap formation in COVID-19 is inhibited by the protein kinase C inhibitor ruboxistaurin. *ERJ Open Res* **8**, (2022).
4. Cui, Y. *et al.* β-carboline alkaloids attenuate bleomycin induced pulmonary fibrosis in mice through inhibiting NF-kb/p65 phosphorylation and epithelial-mesenchymal transition. *J Ethnopharmacol* **243**, 112096 (2019).
5. Trivedi, C. M., Patel, R. C. & Patel, C. V. Homeobox gene HOXA9 inhibits nuclear factor-kappa B dependent activation of endothelium. *Atherosclerosis* **195**, e50–e60 (2007).
6. Jiang, K. *et al.* Barbaloin protects against lipopolysaccharide (LPS)-induced acute lung injury by inhibiting the ROS-mediated PI3K/AKT/NF-κB pathway. *International Immunopharmacology* **64**, 140–150 (2018).
7. Wang, T. & Lu, H. Ganoderic acid A inhibits ox-LDL-induced THP-1-derived macrophage inflammation and lipid deposition via Notch1/PPARγ/CD36 signaling. *Advances in Clinical and Experimental Medicine: Official Organ Wroclaw Medical University* (2021).
8. Buhrmann, C., Brockmueller, A., Mueller, A.-L., Shayan, P. & Shakibaei, M. Curcumin attenuates environment-derived osteoarthritis by Sox9/NF-kB signaling axis. *International Journal of Molecular Sciences* **22**, 7645 (2021).
9. Natarajan, K., Singh, S., Burke, T. R., Grunberger, D. & Aggarwal, B. B. Caffeic acid phenethyl ester is a potent and specific inhibitor of activation of nuclear transcription factor NF-kappa B. *Proceedings of the National Academy of Sciences* **93**, 9090–9095 (1996).

10. Li, M. Y. *et al.* Convallatoxin protects against dextran sulfate sodium-induced experimental colitis in mice by inhibiting NF-κB signaling through activation of PPARγ. *Pharmacological Research* **147**, 104355 (2019).
11. Saber, S., Goda, R., El-Tanbouly, G. S. & Ezzat, D. Lisinopril inhibits nuclear transcription factor kappa B and augments sensitivity to silymarin in experimental liver fibrosis. *International Immunopharmacology* **64**, 340–349 (2018).
12. Baradaran Rahimi, V. *et al.* Carnosol Attenuates LPS-Induced Inflammation of Cardiomyoblasts by Inhibiting NF-κB: A Mechanistic in Vitro and in Silico Study. *Evidence-Based Complementary and Alternative Medicine* **2022**, (2022).
13. Huang, X. *et al.* Dihydroartemisinin attenuates lipopolysaccharide-induced acute lung injury in mice by suppressing NF-κB signaling in an Nrf2-dependent manner. *Int J Mol Med* **44**, 2213–2222 (2019).
14. Choi, M.-K. *et al.* The DPA-derivative 11S, 17S-dihydroxy 7, 9, 13, 15, 19 (Z, E, Z, E, Z)-docosapentaenoic acid inhibits IL-6 production by inhibiting ROS production and ERK/NF-κB pathway in keratinocytes HaCaT stimulated with a fine dust PM10. *Ecotoxicology and Environmental Safety* **232**, 113252 (2022).
15. Tilley, J. W. *et al.* Identification of a Small Molecule Inhibitor of the IL-2/IL-2Rα Receptor Interaction Which Binds to IL-2. *J Am Chem Soc* **119**, 7589–7590 (1997).
16. Nguyen, T. T. M. *et al.* Four new lignans and IL-2 inhibitors from Magnoliae Flos. *Chemical and Pharmaceutical Bulletin* **65**, 840–847 (2017).
17. Leimbacher, M. *et al.* Discovery of Small-Molecule Interleukin-2 Inhibitors from a DNA-Encoded Chemical Library. *Chemistry – A European Journal* **18**, 7729–7737 (2012).
18. Thanhäuser, A. *et al.* Pentoxifylline: a potent inhibitor of IL-2 and IFN-gamma biosynthesis and BCG-induced cytotoxicity. *Immunology* **80**, 151 (1993).
19. Waal, N. D. *et al.* Identification of nonpeptidic small-molecule inhibitors of interleukin-2. *Bioorg Med Chem Lett* **15**, 983–987 (2005).
20. Boumpas, D. T. *et al.* Dexamethasone inhibits human interleukin 2 but not interleukin 2 receptor gene expression in vitro at the level of nuclear transcription. *J Clin Invest* **87**, 1739–1747 (1991).

21. Takahashi, I., Miyaji, H., Yoshida, T., Sato, S. & Mizukami, T. Selective inhibition of IL-2 gene expression by trichostatin A, a potent inhibitor of mammalian histone deacetylase. *J Antibiot (Tokyo)* **49**, 453–457 (1996).
22. Eriksen, K. W. *et al.* Constitutive STAT3-activation in Sezary syndrome: tyrphostin AG490 inhibits STAT3-activation, interleukin-2 receptor expression and growth of leukemic Sezary cells. *Leukemia* **15**, 787–793 (2001).
23. Genovese, M. C. *et al.* A 24-week, randomized, double-blind, placebo-controlled, parallel group study of the efficacy of oral SCIO-469, a p38 mitogen-activated protein kinase inhibitor, in patients with active rheumatoid arthritis. *J Rheumatol* **38**, 846–854 (2011).
24. Nishida, M., Okumura, Y., Sato, H. & Hamaoka, K. Delayed inhibition of p38 mitogen-activated protein kinase ameliorates renal fibrosis in obstructive nephropathy. *Nephrology Dialysis Transplantation* **23**, 2520–2524 (2008).
25. Zhou, C., Shi, X., Huang, H., Zhu, Y. & Wu, Y. Montelukast attenuates neuropathic pain through inhibiting p38 mitogen-activated protein kinase and nuclear factor-kappa B in a rat model of chronic constriction injury. *Anesthesia & Analgesia* **118**, 1090–1096 (2014).
26. Mócsai, A. *et al.* Kinase pathways in chemoattractant-induced degranulation of neutrophils: the role of p38 mitogen-activated protein kinase activated by Src family kinases. *The Journal of Immunology* **164**, 4321–4331 (2000).
27. Schreiber, S. *et al.* Oral p38 mitogen-activated protein kinase inhibition with BIRB 796 for active Crohn's disease: a randomized, double-blind, placebo-controlled trial. *Clinical Gastroenterology and Hepatology* **4**, 325–334 (2006).
28. Xiong, L.-L. *et al.* Administration of SB239063, a potent p38 MAPK inhibitor, alleviates acute lung injury induced by intestinal ischemia reperfusion in rats associated with AQP4 downregulation. *International Immunopharmacology* **38**, 54–60 (2016).
29. Manthey, C. L., Wang, S., Kinney, S. D. & Yao, Z. SB202190, a selective inhibitor of p38 mitogen-activated protein kinase, is a powerful regulator of LPS-induced mRNAs in monocytes. *J Leukoc Biol* **64**, 409–417 (1998).
30. Piao, C. S., Kim, J., Han, P. & Lee, J. Administration of the p38 MAPK inhibitor SB203580 affords brain protection with a wide therapeutic window against focal ischemic insult. *J Neurosci Res* **73**, 537–544 (2003).

31. Newby, L. K. *et al.* Losmapimod, a novel p38 mitogen-activated protein kinase inhibitor, in non-ST-segment elevation myocardial infarction: a randomised phase 2 trial. *The Lancet* **384**, 1187–1195 (2014).
32. Koeberle, S. C. *et al.* Skepinone-L is a selective p38 mitogen-activated protein kinase inhibitor. *Nat Chem Biol* **8**, 141–143 (2012).
33. Schwenger, P. *et al.* Sodium salicylate induces apoptosis via p38 mitogen-activated protein kinase but inhibits tumor necrosis factor-induced c-Jun N-terminal kinase/stress-activated protein kinase activation. *Proceedings of the National Academy of Sciences* **94**, 2869–2873 (1997).
34. Laufer, S. & Lehmann, F. Investigations of SCIO-469-like compounds for the inhibition of p38 MAP kinase. *Bioorganic & Medicinal Chemistry Letters* **19**, 1461–1464 (2009).
35. Bendell, J. C. *et al.* A phase 1 dose-escalation study of checkpoint kinase 1 (CHK1) inhibitor prexasertib in combination with p38 mitogen-activated protein kinase (p38 MAPK) inhibitor ralimetinib in patients with advanced or metastatic cancer. *Investigational New Drugs* **38**, 1145–1155 (2020).
36. Westra, J., Limburg, P. C., de Boer, P. & van Rijswijk, M. H. Effects of RWJ 67657, a p38 mitogen activated protein kinase (MAPK) inhibitor, on the production of inflammatory mediators by rheumatoid synovial fibroblasts. *Annals of the Rheumatic Diseases* **63**, 1453 LP – 1459 (2004).
37. Skripchenko, A., Gelderman, M. P. & Vostal, J. G. P38 mitogen activated protein kinase inhibitor improves platelet in vitro parameters and in vivo survival in a SCID mouse model of transfusion for platelets stored at cold or temperature cycled conditions for 14 days. *Plos one* **16**, e0250120 (2021).
38. Emami, H. *et al.* The effect of BMS-582949, a P38 mitogen-activated protein kinase (P38 MAPK) inhibitor on arterial inflammation: a multicenter FDG-PET trial. *Atherosclerosis* **240**, 490–496 (2015).
39. Ye, N., Ding, Y., Wild, C., Shen, Q. & Zhou, J. Small molecule inhibitors targeting activator protein 1 (AP-1) miniperspective. *J Med Chem* **57**, 6930–6948 (2014).
40. Lee, S.-Y., Lee, K.-S., Yi, S. H., Kook, S.-H. & Lee, J.-C. Acteoside suppresses RANKL-mediated osteoclastogenesis by inhibiting c-Fos induction and NF-κB pathway and attenuating ROS production. *PLoS One* **8**, e80873 (2013).

41. Lee, W.-S., Lee, E.-G., Sung, M.-S. & Yoo, W.-H. Kaempferol inhibits IL-1β-stimulated, RANKL-mediated osteoclastogenesis via downregulation of MAPKs, c-Fos, and NFATc1. *Inflammation* **37**, 1221–1230 (2014).
42. Chu, M., Truumees, I., Mierzwa, R., Patel, M. & Puar, M. S. Sch 56396: a new c-fos proto-oncogene inhibitor produced by the fungus Tolypocladium sp. *The Journal of Antibiotics* **50**, 1061–1063 (1997).
43. Ishida, M. *et al.* T-5224, a selective inhibitor of c-Fos/activator protein-1, improves survival by inhibiting serum high mobility group box-1 in lethal lipopolysaccharide-induced acute kidney injury model. *J Intensive Care* **3**, 1–7 (2015).
44. Darwish, R. S. *et al.* Chemical profiling and unraveling of anti-COVID-19 biomarkers of red sage (Lantana camara L.) cultivars using UPLC-MS/MS coupled to chemometric analysis, in vitro study and molecular docking. *Journal of Ethnopharmacology* **291**, 115038 (2022).
45. Darwish, R. S. *et al.* Comparative metabolomics reveals the cytotoxic and anti-inflammatory discriminatory chemical markers of raw and roasted colocynth fruit (: Citrullus colocynthis L.). *RSC Advances* **11**, 37049–37062 (2021).
46. Louis, K. S. & Siegel, A. C. Cell viability analysis using trypan blue: manual and automated methods. *Methods in molecular biology* **740**, 7–12 (2011).
